# A therapeutic-grade purified exosome system alleviates osteoarthritis by regulating autophagy through the BCL2–Beclin1 axis

**DOI:** 10.1186/s12951-025-03807-y

**Published:** 2025-12-05

**Authors:** Gongyin Zhao, Farbod Yousefi, Ichiro Tsukamoto, Steven Moran, Atta Behfar, Christopher Evans, Chunfeng Zhao

**Affiliations:** 1 Mayo Clinic, Rochester, US; 2 Department of Orthopedics, The Second People’s Hospital of ChangZhou, The Third Affiliated Hospital of Nanjing Medical University, ChangZhou Medical Center, Nanjing Medical University, Changzhou, China

**Keywords:** Exosomes, Autophagy, Apoptosis, BCL2, Beclin-1, Cartilage regeneration, Osteoarthritis, Nanomedicine, Joint degeneration, Therapeutic delivery system

## Abstract

**Background:**

Osteoarthritis is a common and progressive degenerative joint disorder marked by cartilage degradation, subchondral bone remodeling, and chondrocyte apoptosis. Autophagy, a tightly regulated intracellular degradation process, is essential for maintaining chondrocyte homeostasis. Dysregulated autophagy can contribute to cartilage degeneration by disrupting the balance between cellular survival and death. The B-cell lymphoma 2 (BCL2) protein plays a dual role by inhibiting autophagy via its interaction with Beclin-1 while simultaneously suppressing apoptosis. This study aimed to investigate whether a therapeutic-grade purified exosome system derived from human plasma can modulate autophagy through regulation of BCL2 signaling, reduce chondrocyte apoptosis, and prevent osteoarthritis progression.

**Results:**

In vitro experiments demonstrated that exosome treatment increased autophagic activity and reduced apoptosis in both immortalized and osteoarthritic human chondrocytes. Mechanistic analysis revealed that exosomes downregulated BCL2 expression, disrupted the BCL2–Beclin-1 complex, and enhanced the expression of autophagy-related proteins LC3 and Beclin-1. Overexpression of BCL2 reversed these effects and led to impaired autophagic flux and elevated apoptosis, particularly in osteoarthritic chondrocytes. In a rat model of surgically induced osteoarthritis, intra-articular injection of the exosome product mixed with hyaluronic acid improved gait parameters, reduced mechanical pain sensitivity, and preserved cartilage architecture and subchondral bone structure. Histological and molecular analyses confirmed reduced chondrocyte apoptosis and elevated autophagic activity in exosome-treated joints, along with decreased BCL2 expression and complex formation with Beclin-1.

**Conclusions:**

This study demonstrates that a therapeutic-grade exosome formulation can alleviate osteoarthritis by restoring the balance between autophagy and apoptosis through modulation of the BCL2–Beclin-1 signaling axis. These findings highlight the potential of exosome-based nanotherapeutics as a novel disease-modifying treatment strategy for degenerative joint disorders.

**Graphical Abstract:**

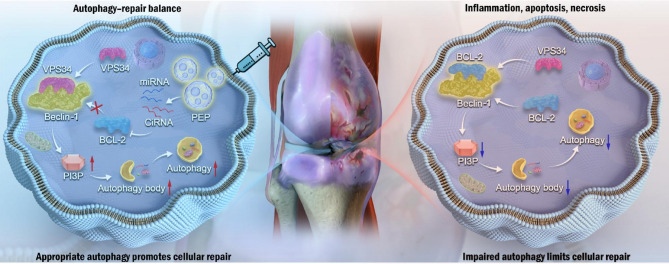

**Supplementary Information:**

The online version contains supplementary material available at 10.1186/s12951-025-03807-y.

## Background

Osteoarthritis (OA) is a chronic degenerative joint condition characterized by pain, stiffness, and progressive loss of joint function [1]. Its increasing prevalence has led to substantial social and healthcare burdens [2]. Despite extensive research, effective prevention and treatment methods remain elusive. Current approaches primarily revolve around symptom management, including pharmaceutical interventions and total joint replacement surgeries [3, 4].

Apoptosis, a controlled cell death process, plays a significant role in OA’s development [5, 6]. In the early stages of OA, chondrocyte apoptosis leads to cartilage loss and joint degeneration [7], triggered by mechanical stress, inflammation, and oxidative stress [8]. Targeted inhibition of specific apoptotic pathways can reduce cartilage degradation and improve joint function in OA [9]. Autophagy, a vital cellular process, maintains homeostasis by recycling damaged components [10, 11]. Autophagy dysregulation is associated with OA [12, 13], with its effects ranging from promoting apoptosis to providing cytoprotective function depending on the upregulated level of autophagy. Excessive autophagy can trigger apoptosis, while appropriate elevation can provide positive effects by removing toxic proteins and damaged organelles, reducing inflammation and decreasing chondrocyte apoptosis. Thus, properly regulated autophagy has the potential for OA treatment strategy [12, 13].

Exosomes, vital for intercellular communication, influence autophagy by carrying cargoes like microRNAs and autophagy-related proteins [14, 15]. Beyond osteoarthritis, accumulating evidence indicates that the crosstalk between autophagy and exosome pathways contributes to disease progression and tissue repair in cancer, ischemia, and cardiovascular disorders [16–19]. Although recent studies have highlighted the role of exosomes in regulating chondrocyte viability and matrix metabolism, the therapeutic efficacy and mechanistic targets of clinically applicable exosome formulations in osteoarthritis remain poorly defined. Whether exosomes can modulate the BCL2–Beclin1 signaling axis to restore autophagic homeostasis in chondrocytes has not been explored.

The PEP vials are meticulously produced by Rion LLC in collaboration with Mayo Clinic’s Advanced Product Incubator (API) within the Center for Regenerative Medicine. Operating under FDA-regulated cGMP standards, API ensures the creation of a high-quality medical-grade product. The process involves acquiring expired human donor plasma, isolating exosomes through separation, filtration, and centrifugation, and encapsulating them per US Patent 20160324A1. PEP was manufactured by Rion LLC under FDA-regulated cGMP standards and lyophilized for room-temperature storage. This process showcases cutting-edge biotechnology’s potential to transform regenerative medicine. PEP has shown promise in tissue repair, included tendons, skin, nervous system, etc [20–25]., but its potential role in cartilage protection and osteoarthritis intervention, especially through modulation of autophagy–apoptosis signaling, remains unknown.

We hypothesized that PEP could restore the disrupted autophagy–apoptosis balance in osteoarthritic chondrocytes by downregulating BCL2 expression, disrupting the inhibitory BCL2–Beclin1 complex, and thereby activating protective autophagic pathways while limiting chondrocyte death.

To test this hypothesis, we designed a comprehensive study with the following objectives:

To assess the effects of PEP on autophagic flux and apoptosis in both healthy and osteoarthritic human chondrocytes in vitro; To determine whether PEP modulates chondrocyte autophagy via regulation of BCL2–Beclin1 signaling; To evaluate the in vivo efficacy of PEP delivered intra-particularly in a rat model of surgically induced osteoarthritis, focusing on functional improvement, histological preservation, and molecular markers of cartilage health. This study provides new insights into the mechanistic and therapeutic potential of clinical-grade exosome systems in the context of osteoarthritis and supports the development of exosome-based nanotherapeutics as disease-modifying interventions in degenerative joint diseases.

## Methods

### Identification of protein markers on the surface of PEP

One vial of PEP (lot # 23001 A) was purified using the ExoEasy Maxi Kit (Cat#: 76064; Qiagen^®^, Hilden, Germany). The PEP cake powder was resuspended in 2mL RNase-free water, filtered (cat# 16592; Minisart^®^ Syringe Filter, Surfactant-free Cellulose Acetate Pore Size 0.8 μm; Gottingen, Germany), and processed per Qiagen’s protocol. Protein concentration was determined using the BCA method (cat# 23225; Pierce™, ThermoFischer©, Waltham, MA) yielding 2.4 µg/µL. For electrophoresis, 15 µg of protein and 8µL molecular marker (cat#: LC5925, SeeBlue™ Plus2 ladder, ThermoFischer©, Waltham, MA) were loaded onto a separating gel (cat#: NW04122Box, Invitrogen™ Bolt™ 4–12% Bis-Tris Plus gel, ThermoFischer©, Waltham, MA) with running buffer (cat#: NP0002, NuPage™ MES Running Buffer, ThermoFischer©, Waltham, MA), then transferred to a PVDF membrane (cat#88520, ThermoFischer©, Waltham, MA). After blocking (5% nonfat dry milk in 1XTBST) for 1 h at room temperature, the membrane was incubated overnight at 4 °C with primary antibodies: anti-rabbit CD63 (cat#: MAB50482; dilution 1:10,000, R&D systems Inc., Minneapolis, MN) and anti-rabbit CD9 (cat#: ab236630; dilution 1:1000, Abcam©, Cambridge, UK). Following washing and incubation with HRP-linked secondary antibody (anti-rabbit HRP, cat#: HAF008; dilution 1:5000, R&D systems Inc., Minneapolis, MN), protein visualization was done using the enhanced chemiluminescent kit (cat #: A38556, Supersignal™ West Atto, ThermoFischer©, Waltham, MA) via iBright CL1500 system. After detection, the membrane was re-blocked and probed for beta-actin expression (anti-rabbit beta-actin, cat#: ab8227; dilution 1:2800, Abcam©, Cambridge, UK).

### Cell cultures and transfection

Experiments involving the use of human samples were reviewed and approved by the Institutional Review Board at the Mayo Clinic (IRB no. 13–005619), and written informed consent was obtained from all participants. The immortalized human chondrocyte cell line C28/I2 was kindly provided by E Ferreira (University of Arkansas for Medical Sciences, AR, USA). Human primary chondrocytes were isolated from cartilage explants of a single patient (female, 61 years of age, diagnosed with knee OA, Kellgren–Lawrence grade III) undergoing total knee replacement surgery, following established protocols [26]. Both cell lines were cultured in DMEM/Hams F-12 supplemented with 10% FBS and 1% antibiotic-antimycin at 37 °C with 5% CO2. Primary OA chondrocyte experiments were conducted after the second in vitro passage.

BCL2 plasmid was extracted by plasmid extraction kit (Chargeswitch™ Pro Filter Plasmid Mini Kit). For transfection, 15 pmol of RNA oligomer solution was mixed with 100 µL of Opti-MEM™ medium (Invitrogen, Carlsbad, CA). To promote transfection, 0.6 µL of Lipofectamine^®^ 3000 (Invitrogen) solution was added to the mixture. The resulting complex was incubated for an additional 20 min at room temperature.

### Detection of chondrocyte apoptosis

Chondrocyte apoptosis was assessed using the Apoptosis Kit (4440; Incucyte) according to the manufacturer’s instructions. Briefly, C28/I2 cells were seeded at 10,000 cells per well in a 96-well plate and incubated for 48 h at 37 °C with 5% CO2. Subsequently, cells were exposed to a complete medium with IL-1β (10 ng/ml, #200-01B, Peprotech, NJ, USA) and various drugs (including PEP at 5%, rapamycin, chloroquine, etc.) for an additional 48 h. Apoptotic chondrocytes were detected using Incucyte 4440 (Caspase-3/7 Green Dye, 1:1000) in DMEM/F-12 medium, and real-time fluorescence images were captured and analyzed using the Incucyte system.

### Autophagic flow assay in chondrocytes

Cells from different experimental groups were treated with different drugs for 48 h and then subjected to immunofluorescence. Primary antibodies used were LC3-B (0.1 µg/ml, #PA1-46286, Invitrogen) and Beclin-1 (1:100, #MA5-25480, Invitrogen). Secondary antibodies included goat anti-rabbit Alexa Fluor 633 (4 µg/ml, #A-21070, Invitrogen) and donkey anti-mouse Alexa Fluor 488 (0.2 µg/ml, #A-21202, Invitrogen) Nuclei were stained with 0.1 µg/ml Hoechst 33,342 for 10 min.

### Animal study design and rat OA model

Forty-eight Sprague-Dawley rats (adult females, 4–5 months of age, weighing 562–702 g). The rats were divided into two groups, one for treatment (24 rats) and the other for prevention (24 rats). Each group of rats was further randomized into three groups: control group, HA group, and HA@PEP group (*n* = 8 in each group). We chose the right side as the surgical side **(**Fig. 4A**)**. After successful anesthesia, the anterior cruciate ligament was cut, and the medial meniscus was removed. **(**Fig. 4B, C**).** After successful anesthesia, the anterior cruciate ligament was cut, and the medial meniscus was removed.

**(**Fig. 4B, C**).** Osteoarthritis was induced using this combined anterior cruciate ligament transection (ACLT) and destabilization of the medial meniscus (DMM) model in rats, as previously described [27–29]. For the prophylaxis group, we performed intra-articular injections using a 25G needle syringe under ultrasound guidance after suturing the skin, 50ul each time, once a week for four weeks **(**Fig. 4D, E**).** For the treatment part, the rats were injected in the joint cavity, in the same way, starting from the 8th week after surgery. All rats were euthanized by CO2 asphyxiation at the end of the injections (four in each group for histological analysis and four for PCR and Western blot detection).

### Preparation of HA@PEP and release test

We mixed 1 vial of PEP with 1 ml of PBS and then mixed the 100% PEP solution with different amounts of hyaluronic acid (hylan G-F 20, Synvisc, USA) to obtain different concentrations of HA@PEP solution (10%, 20%, and 50% HA@PEP). We slowly injected 2 ml of saline into each vial of HA@PEP mixture, and the liquid was divided into two layers without stirring. The solutions were placed in a 37 °C incubator overnight. The next day, the upper saline layer of each HA@PEP concentration was collected for NanoSight particle analysis. The particles were analyzed for seven consecutive days and the number of particles obtained was compared to the number of particles in the 5% PEP solution.

### Gait analysis and von Frey test

At specific time points, we used the DigiGait rodent treadmill system (Mouse Specifics, Inc.) to assess various gait parameters, coordination, balance, and motor function objectively and quantitatively. During the assessment period, all mice were asked to walk on a DigiGait treadmill for at least 3 s continuously, maintaining a constant speed of 15 cm/s. Gait was analyzed using the DigiGait analysis system. Mechanical nociception in the hind paw was assessed using the von Frey test [30–33]. On the day of the actual assessment, the rats were given a 20-minute acclimatization period in the test cage prior to the initiation of the test. During the evaluation, von Frey filaments (Touch Test, U.S.) with forces ranging from 2 to 8 G were systematically applied in ascending order to the plantar surface of each hind paw. Each paw underwent five repetitions of the filament application. The filament force that induced nociceptive behavior was recorded for three out of the five applications, specifically focusing on the observable behaviors of paw licking, prolonged paw withdrawal, and flapping/shaking of the paw. The withdrawal thresholds for both the left and right hind paws were averaged for subsequent statistical analysis to ensure precise measurements. In the prevention cohort, gait parameters at week 0 served as the pre-surgical baseline to confirm comparability across groups. In the treatment cohort, animals were randomized into groups at 8 weeks post-surgery, when OA pathology was established, to specifically evaluate therapeutic efficacy in an established disease setting rather than preventive effects.

### Micro-CT

After the rats were executed, the distal femur and proximal tibia underwent high-resolution scanning with the SkyScan1276 system (SkyScan1276, Micro Photonics Inc, PA, U.S.). The image pixel size was 40 μm, and the exposure time was 140ms. X-ray energy parameters were carefully set to 200 mA and 50 kVp. After the acquisition of 2D image slices, a well-defined region of interest was consistently delineated, and subsequently a three-dimensional (3D) reconstruction was generated for comprehensive analysis. The reconstruction process required the application of specific parameters, i.e., Gaussian = 0.8, s = 1, and threshold = 163, to ensure the accuracy and reliability of the overall analysis. Using micro-CT scans, meticulous examinations were performed to assess important parameters including bone volume/tissue volume ratio (BV/TV), bone trabeculae number (Tb. N), bone trabeculae thickness (Tb. Th), and changes in 3D bone structure.

### Quantitative real-time PCR

Cell clusters were digested with trypsin and obtained for further analysis, and tissues were frozen in liquid nitrogen and then ground. cDNA synthesis was performed using the iScript cDNA Synthesis Kit (Bio-Rad Laboratories) and 1 µg of total RNA was reverse transcribed to complementary DNA using the Thermo Script RT Kit (Invitrogen). cDNA was synthesized in triplicate and used for Real-time PCR analysis to quantitatively assess gene expression levels. GAPDH was used as an internal control for numerical normalization. Relevant genes were detected using specific primers, including apoptosis-related genes (Bax, Caspase 3) and autophagy-related genes (LC3-B, Beclin-1), as well as the apoptosis- and autophagy-related gene Bcl-3. Primer sequences are shown in Supplementary Table 1.

### Western blot and co-immunoprecipitation

Total proteins were extracted from C28/I2 and OA chondrocytes, separated by electrophoresis with 4–12% Bis-Tris Plus (#22042712. Invitrogen), and then transferred to polyvinylidene fluoride membranes (#IPVH00010, Millipore, USA). After routine closure the membrane was placed on LC3-B (1:1000, #PA1-46286, Invitrogen), Bcl-2 (1ug/ml, #138800, Invitrogen) primary antibody. Secondary antibodies were horseradish peroxidase-conjugated goat anti-rabbit IgG (1:5000, #7074S, cell signaling technology) and goat anti-mouse IgG (1:5000, #7076S, cell signaling technology). Proteins bound to Bcl-2 (1 µg/ml, #138800, Invitrogen) antibody were isolated using Pierce™ Crosslink Magnetic IP/Co-IP kit (Invitrogen). This was then detected with an anti-Beclin-1 primary antibody (1:2000, #PA1-16857, Invitrogen) and goat anti-rabbit IgG secondary antibody (1:5000, #7074S, Cell Signaling Technology). For IgG negative controls, species-matched normal IgG was used instead of primary antibody. TrueBlot secondary antibodies (Rockland) were applied to avoid detection of IgG heavy and light chains. All Co-IP experiments were performed in triplicate (*n* = 3 independent biological replicates). All signals were detected by an iBright 1500 Imaging System (Invitrogen, USA) and analyzed for protein density. quantification.

### Immunofluorescence

Rat knee tissue sections were subjected to a meticulous deparaffinization process, involving sequential immersion in xylene followed by graded ethanol solutions with varying concentrations ranging from 100% to 50%. Subsequently, the sections were treated with 5% bovine serum albumin (BSA) to block non-specific binding sites for 1 h at room temperature. Following this, the tissue sections were incubated overnight at 4 °C with primary antibodies, namely LC3-B (0.1 µg/ml, #PA1-46286, Invitrogen) and Bcl-2 (1:100, #138800, Invitrogen). Upon completion of the overnight incubation with the primary antibodies, the tissue sections were exposed to secondary antibodies, including Goat anti-Rabbit Alexa Fluor 633 (4 µg/ml, #A-21070, Invitrogen) and Donkey anti-Mouse Alexa Fluor 488 (0.2 µg/ml, #A-21202, Invitrogen), for 1 h at room temperature. To visualize cell nuclei, the sections were stained with 0.1 µg/ml Hoechst 33,342 for 10 min. Fluorescence images were captured from randomly selected fields of view using a confocal microscope (LSM780, Zeiss, Germany). For cellular immunofluorescence, the cells were initially fixed with 4% paraformaldehyde (PFA) and subsequently permeabilized using 0.1% Triton X100 for 15 min. The remaining steps in the cellular immunofluorescence staining process were consistent with the procedures employed for histologic immunofluorescence staining.

### Histology analysis

After rats were sacrificed, their affected knee joints were fixed in 4% paraformaldehyde (PFA) for 3 days, and the samples then underwent decalcification in 10% EDTA (#1340, Biofroxx) solution for 2 months. The decalcified tissues were then embedded in paraffin blocks and cut into 5-µm coronal slides by a microtome (Thermo, Germany). Tissue sections from each experimental group were stained by hematoxylin and eosin (H&E) (#C0105S Beyotime, Shanghai, China), safranin O/fast green (#G1371, Solarbio, Beijing, China), and TUNEL (Click-iT™ TUNEL Colorimetric IHC Detection Kit, Thermo Fisher #C10625). The Osteoarthritis Research Society International (OARSI) scoring and synovitis scoring22 were assessed by two individuals who were ignorant of the animals’ treatment. All tissue sections were randomly coded by a third investigator prior to scoring to ensure blinding. The OARSI grading system was applied, and the mean value of the two observers’ scores was used for analysis. In cases where the two scores differed by more than one grade, a consensus score was reached after joint review. To minimize the impact of technical artifacts such as tissue detachment during decalcification, sections with extensive damage were excluded from analysis. Quantification (including OARSI and TUNEL scoring) was therefore restricted to intact regions of cartilage and subchondral bone, and statistical conclusions were derived from systematic evaluation across multiple animals and serial sections.

### Statistical analysis

Statistical analysis Data were presented as mean (SD). Each trial was performed independently at least 3 times. Kruskal-Wallis one-way analysis of variance test with Dunn test was used for determining statistical significance for 2-group comparisons and multiple-group comparisons, respectively. Statistical comparisons between 2 groups were analyzed by nonpaired Student t-test or Mann-Whitney test. All statistical tests were performed using GraphPad Prism 8 (GraphPad Software Inc). Results with asterisks were considered statistically significant (*P < 0.05, **P < 0.01, ***P < 0.001, ****P < 0.0001).

## Results

### PEP elicited autophagic responses in both C28/I2 and osteoarthritic chondrocytes, which in turn contributed to a reduction in apoptosis, highlighting the cytoprotective role of autophagy in these cells

We first conducted a preliminary screening and identified that 5% PEP exhibited the most potent inhibitory effect on interleukin-1β (IL-1β)-induced apoptosis in C28/I2 chondrocytes (Supplementary Fig. 1). Fluorescence imaging revealed that within 24 h of co-incubation, PEP was progressively internalized by chondrocytes and predominantly localized in the perinuclear region (Supplementary Fig. 2A–C).

**Fig. 1:**
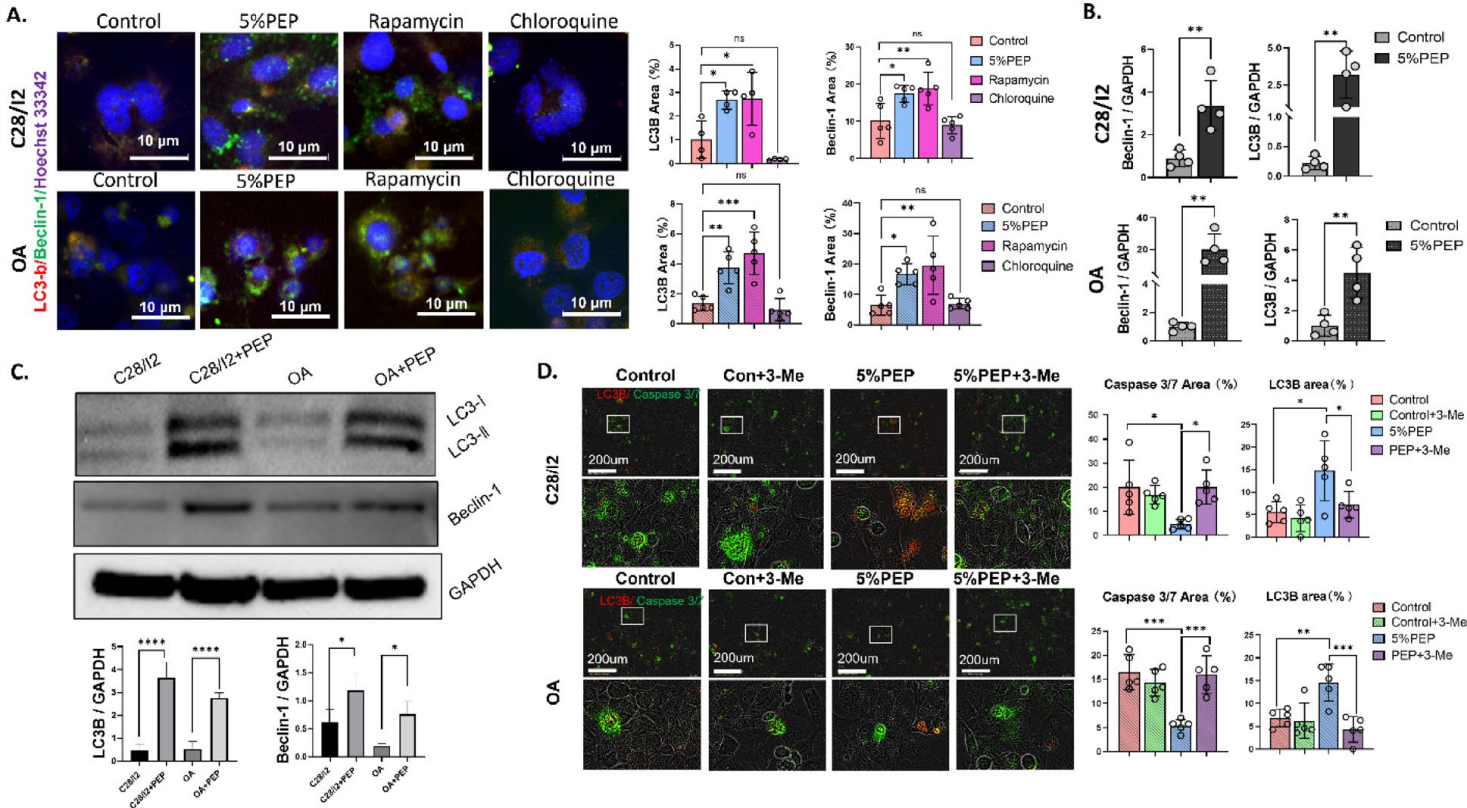
PEP activates autophagy and reduces apoptosis in C28/I2 and osteoarthritic chondrocytes. **A** Immunofluorescence analysis of LC3B (red) and Beclin-1 (green) expression in C28/I2 and OA chondrocytes following PEP treatment. Rapamycin was used as a positive control for autophagy induction, and chloroquine as a negative control for autophagy inhibition. **B** Quantitative real-time PCR analysis showing increased mRNA expression of LC3B and Beclin-1 in PEP-treated cells compared to the control group. **C** Western blot analysis confirming elevated protein expression levels of LC3B and Beclin-1 in both cell types upon PEP treatment. **D** IncuCyte live-cell imaging analysis demonstrating increased expression of LC3B and decreased levels of caspase-3/7 in the PEP group, with opposite effects observed upon 3-methyladenine (3-MA) treatment.Data are presented as mean ± standard deviation (SD). *p < 0.05, **p < 0.01, ***p < 0.001, ****p < 0.0001.

To investigate the role of PEP in autophagy regulation, we performed autophagy flow cytometry and observed a significant upregulation of LC3B and Beclin-1 protein levels in both C28/I2 and osteoarthritic chondrocytes following PEP treatment, compared to untreated controls **(**Fig. 1A, Supplementary Fig. 3). Consistently, quantitative PCR analysis demonstrated elevated transcript levels of LC3B and Beclin-1 in the PEP-treated group **(**Fig. 1B**)**, which was further supported by Western blot results showing enhanced protein expression in both cell types **(**Fig. 1C**).**

To further elucidate the relationship between autophagy and apoptosis, we assessed the expression of LC3B and caspase-3/7. PEP treatment increased LC3B expression while reducing caspase-3/7 levels relative to the control group. Notably, pharmacological inhibition of autophagy using 3-methyladenine (3-MA) reversed these effects, suppressing LC3B expression and increasing caspase-3/7 levels **(**Fig. 1D**)**. These findings were corroborated by transcriptional data from PCR analysis (Supplementary Fig. 4), collectively indicating that the cytoprotective effect of PEP is mediated, at least in part, through autophagy induction.

### PEP restores autophagic flux by downregulating BCL2 and attenuating BCL2–Beclin1–mediated inhibition in chondrocytes

**Fig. 2:**
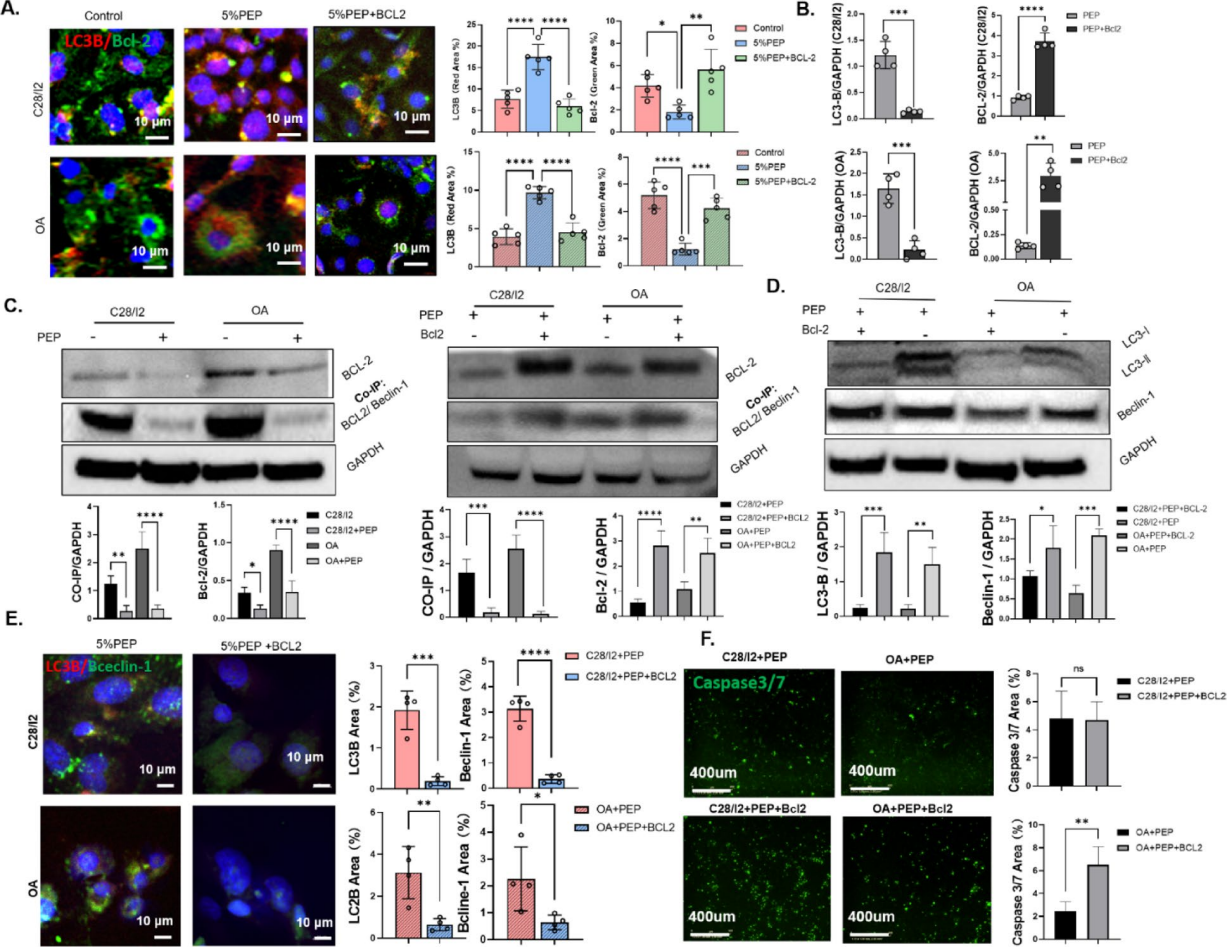
PEP promotes chondrocyte autophagy by downregulating BCL2 and disrupting the BCL2–Beclin1 interaction. **(A)** Immunofluorescence staining of LC3B (red) and BCL2 (green) in chondrocytes with or without BCL2 overexpression following PEP treatment. **(B)** Quantitative PCR analysis of BCL2 mRNA expression in chondrocytes before and after BCL2 transfection. **(C)** Western blot and co-immunoprecipitation analysis showing changes in BCL2 expression and BCL2–Beclin1 complex formation following BCL2 overexpression. **(D)** Western blot quantification of LC3B and Beclin-1 protein expression following BCL2 overexpression in PEP-treated cells. **(E)** Immunofluorescence-based assessment of autophagic flux in C28/I2 and osteoarthritic chondrocytes after BCL2 transfection. **(F)** Apoptosis levels in C28/I2 and osteoarthritic chondrocytes following BCL2 overexpression, as evaluated by IncuCyte analysis. Data are presented as mean ± SD. *p < 0.05, **p < 0.01, ***p < 0.001, ****p < 0.0001.

Immunofluorescence analysis revealed that PEP treatment significantly downregulated BCL2 protein expression and upregulated LC3B levels compared to the control group. To further validate the role of BCL2, chondrocytes were transfected with a BCL2-overexpression plasmid 24 h after PEP treatment. This intervention restored BCL2 expression and subsequently reduced LC3B levels, as confirmed by immunofluorescence and quantitative PCR analyses **(**Fig. 2A, Supplementary Fig. 5 A; Fig. 2B**)**.

Western blot and co-immunoprecipitation assays consistently demonstrated reduced BCL2 expression and decreased formation of the BCL2–Beclin-1 complex following PEP exposure, whereas BCL2 overexpression led to elevated levels of both proteins. Both unidirectional (IP: Beclin-1; IB: BCL-2) and reciprocal (IP: BCL-2; IB: Beclin-1) Co-IP confirmed that PEP treatment reduced the interaction between BCL-2 and Beclin-1. Densitometric quantification of Co-IP bands (normalized to input) from three independent experiments further supported this finding (Fig. 2C, Supplementary Fig. 8 A). Moreover, BCL2 overexpression following PEP treatment significantly suppressed autophagy-related proteins LC3B and Beclin-1 (Fig. 2D, Supplementary Fig. 8B), which was accompanied by a marked decrease in autophagic flux in both C28/I2 and osteoarthritic chondrocytes, as assessed by immunofluorescence (Fig. 2E, Supplementary Fig. 5B).

While apoptosis levels remained unchanged in C28/I2 cells, BCL2 overexpression following PEP treatment led to a significant increase in apoptosis in osteoarthritic chondrocytes **(**Fig. 2F**)**. These findings suggest that PEP promotes autophagy and limits apoptosis in OA chondrocytes, at least in part, by downregulating BCL2 expression and disrupting the inhibitory BCL2–Beclin-1 interaction.

### PEP-induced autophagy exerts cytoprotective effects without inducing autophagy-associated cell death

To further evaluate the magnitude and characteristics of PEP-induced autophagy, we conducted a comparative analysis with rapamycin-induced autophagy and starvation-induced autophagy (4-hour serum deprivation). The three models exhibited distinct autophagic profiles: rapamycin induced the strongest autophagic response, followed by PEP, while starvation-induced autophagy was the least robust.

**Fig. 3:**
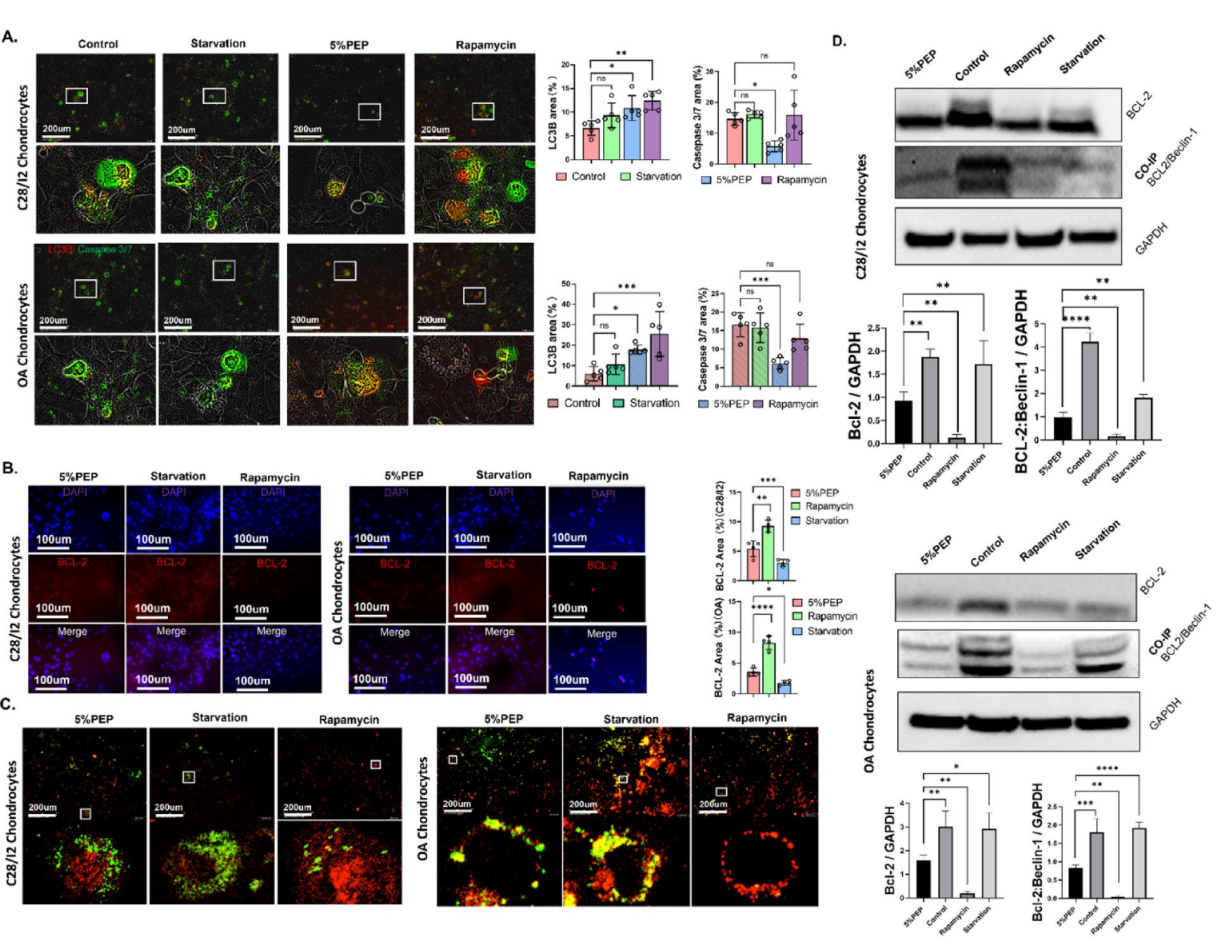
Comparative analysis of autophagy and BCL2–Beclin-1 pathway modulation by different autophagy inducers. **(A)** IncuCyte live-cell imaging analysis of autophagy (LC3B) and apoptosis (caspase-3/7) marker expression in chondrocytes treated with rapamycin, PEP, or starvation. **(B)** Immunofluorescence staining showing differential suppression of BCL2 expression by the three autophagy inducers. **(C)** Immunofluorescence-based analysis of BCL2–Beclin-1 complex formation. BCL2 is shown in green; Beclin-1 in red. **(D)** Western blot and co-immunoprecipitation results depicting BCL2 expression and BCL2–Beclin-1 complex levels across treatment groups. Data are presented as mean ± SD. *p < 0.05, **p < 0.01, ***p < 0.001, ****p < 0.0001.

Caspase-3/7 expression was significantly elevated in both the rapamycin and starvation groups compared to the PEP group, suggesting a potential association between excessive autophagy and apoptosis under those conditions **(**Fig. 3A**)**. Immunofluorescence analysis of BCL2 transcription revealed a graded inhibitory pattern: rapamycin led to the most pronounced downregulation, followed by PEP, with starvation exerting only minimal suppression **(**Fig. 3B**)**.

Consistent with these findings, immunofluorescence and co-immunoprecipitation assays demonstrated that rapamycin most effectively suppressed BCL2 protein levels and disrupted the BCL2–Beclin-1 complex, whereas starvation had the least impact. PEP induced a moderate but significant reduction in both BCL2 expression and complex formation **(**Fig. 3C and D**)**. The binding affinity of the BCL2–Beclin-1 complex was strongest in the starvation group and weakest in the rapamycin group, with PEP exhibiting intermediate effects. These results were further supported by PCR analysis (Supplementary Fig. 6).

Collectively, these data suggest that while rapamycin induces a potent autophagic response, it is accompanied by increased apoptotic signaling, unlike PEP, which triggers autophagy in a more controlled, cytoprotective manner.

### Therapeutic exosome intervention improves functional outcomes in ACLT-DMM–induced osteoarthritis in rats

**Fig. 4:**
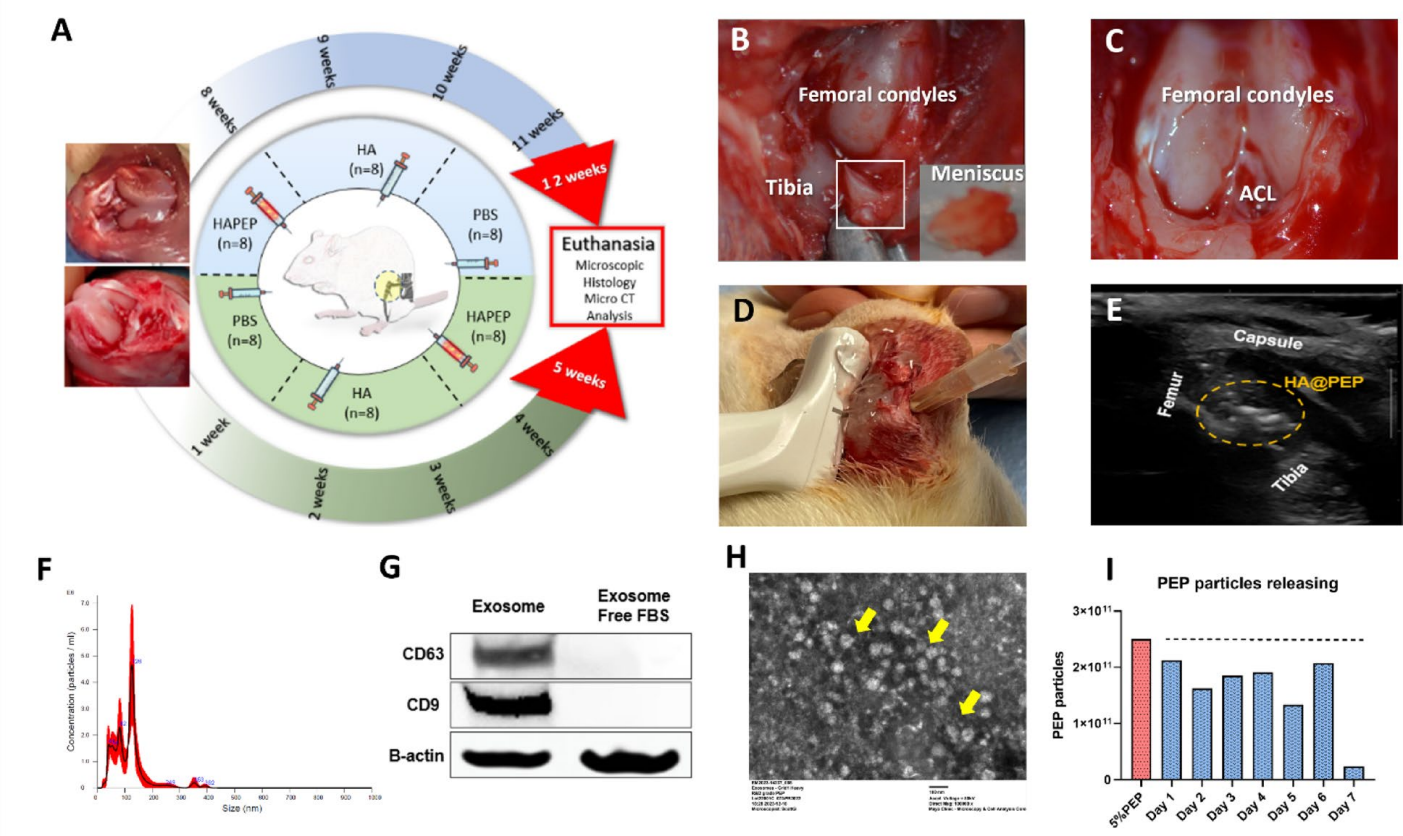
Surgical modeling, ultrasound-guided injection, and characterization of the HA@PEP formulation. **(A)** Schematic of animal group allocation and experimental timeline. **(B)** Medial meniscectomy performed via arthrotomy of the right knee. **(C)** Anterior cruciate ligament transection of the right knee to induce joint instability. **(D)** Ultrasound-guided intra-articular injection into the right knee joint cavity. **(E)** Real-time ultrasound monitoring of drug administration. **(F)** Nanoparticle tracking analysis (NTA) showing particle size distribution and concentration of PEP. **(G)** Western blot characterization of exosome surface markers. **(H)** Scanning electron microscopy (SEM) image of PEP particles (indicated by yellow arrows). **(I)** In vitro release profile showing the daily release of PEP particles from a 50% HA@PEP formulation over one week

To evaluate the release characteristics of PEP from the delivery matrix, we performed particle quantification assays. The daily release of exosome particles from a mixture of 1 mL of 100% hyaluronic acid (HA) and 1 mL of 100% PEP solution was comparable to that observed in a standard 5% PEP solution, amounting to approximately 2.5 × 10¹¹ particles **(**Fig. 4F–I**)**. Based on these results, the effective concentration of PEP in the final HA@PEP formulation was determined to be 50%.

**Fig. 5:**
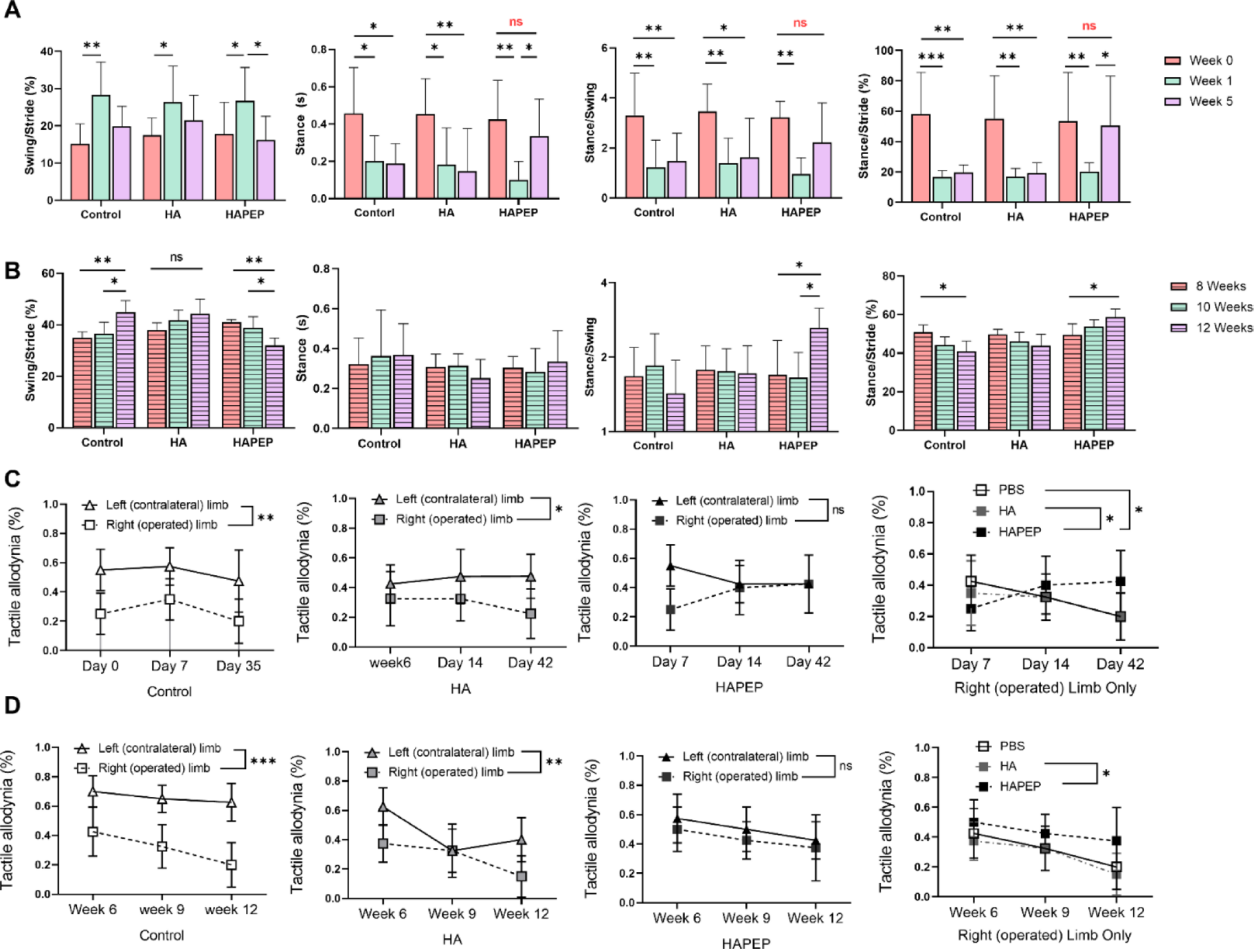
Functional assessment of locomotion and pain sensitivity in OA rats following HA@PEP treatment. **(A)** Quantitative gait analysis in the prevention group showing swing time, stance time, and percentage stance of the right hindlimb over time. **(B)** Gait performance in the treatment group following intra-articular injection of HA@PEP compared to control and HA groups. **(C)** Von Frey test results in the prevention group demonstrating mechanical withdrawal threshold changes in the right hindlimb. **(D)** Von Frey test in the treatment group showing reduced tactile allodynia following HA@PEP administration. Data are presented as mean ± SD. *p < 0.05, **p < 0.01, ***p < 0.001, ****p < 0.0001

In the prevention group, gait analysis revealed a significant reduction in swing time of the right hindlimb within the first week following HA@PEP injection. This improvement was not observed in either the control or HA-only groups. Furthermore, the PEP-treated rats showed progressive restoration of both stance and swing phases during the postoperative period, approaching baseline (preoperative) levels. The percentage of stance time also increased significantly compared to the first postoperative week, with no statistical difference from preoperative values **(**Fig. 5A**)**.

In the treatment group, although there was no statistically significant difference in absolute stance time across the three groups, distinct trends were observed. The HA@PEP group exhibited a gradual decrease in swing phase percentage and a significant increase in stance phase percentage, while the control group showed minimal changes. These findings suggest that both prophylactic and therapeutic administration of PEP effectively alleviated pain and improved functional weight-bearing in the affected limb **(**Fig. 5B**)**.

Moreover, results from the Von Frey test demonstrated that four weeks of HA@PEP treatment significantly reduced tactile allodynia in the right hindlimb. The mechanical withdrawal threshold was restored to levels comparable to the contralateral (left) limb and was significantly different from both the control and HA groups **(**Fig. 5C, D**)**.

Collectively, these data indicate that intra-articular administration of HA@PEP improves locomotor function and alleviates pain in rats with ACLT-DMM–induced osteoarthritis, supporting its potential for both preventive and therapeutic application.

### PEP alleviates osteoarthritis-induced degeneration of cartilage and subchondral bone

**Fig. 6:**
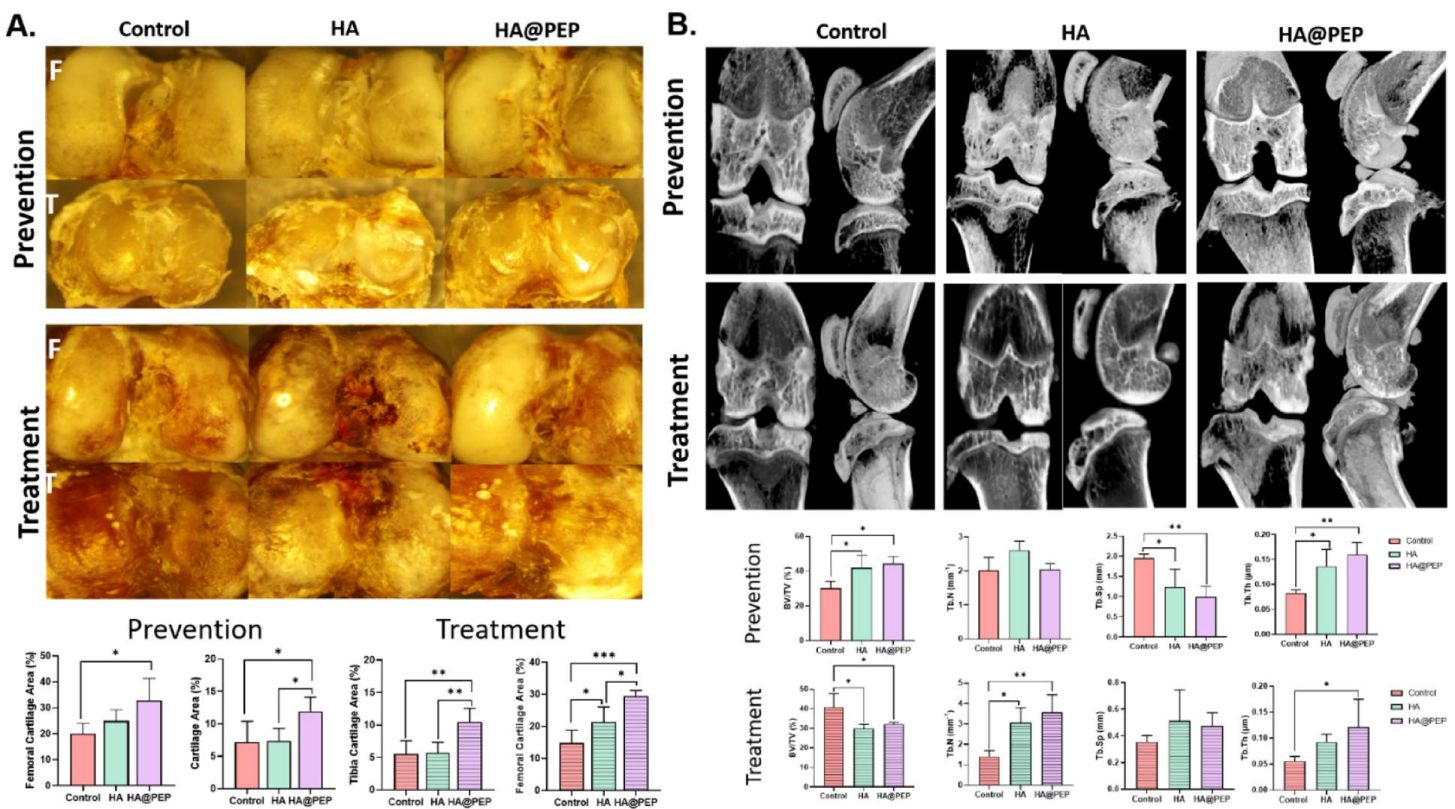
Macroscopic and microstructural evaluation of cartilage and subchondral bone in osteoarthritic knee joints. **(A)** Representative gross images of rat knee joint surfaces from each group showing cartilage integrity and macroscopic degeneration. **(B)** Micro-computed tomography (micro-CT) analysis of the distal femur and proximal tibia demonstrating subchondral bone parameters, including bone volume fraction (BV/TV), trabecular number, and trabecular thickness. Data are presented as mean ± SD. *p < 0.05, **p < 0.01, ***p < 0.001, ****p < 0.0001

In the prevention phase, quantitative analysis of the femoral condyles and tibial plateau showed that HA@PEP treatment preserved a significantly larger cartilage area compared with the control and HA groups. In the treatment phase, cartilage preservation was also evident, with the HA@PEP group showing the most pronounced effect **(**Fig. 6A**).**

Micro-CT analysis of the subchondral bone demonstrated that, in the prevention phase, the HA@PEP group had a higher bone volume fraction (BV/TV) relative to the control and HA groups. Trabecular thickness was significantly increased, while the total trabecular area was reduced, indicating early-stage structural reorganization **(**Fig. 6B**).** In the treatment phase, BV/TV was lower in the HA@PEP group than in the other groups, but both trabecular number and thickness were increased, suggesting active bone remodeling. Excessive increases in trabecular number and thickness were observed in the control and HA groups, consistent with subchondral bone sclerosis. In contrast, the HA@PEP group exhibited a more balanced microarchitecture, indicating that HA@PEP may suppress abnormal bone remodeling associated with OA progression.

### PEP alleviates osteoarthritis by downregulating BCL2 and promoting protective autophagy

**Fig. 7:**
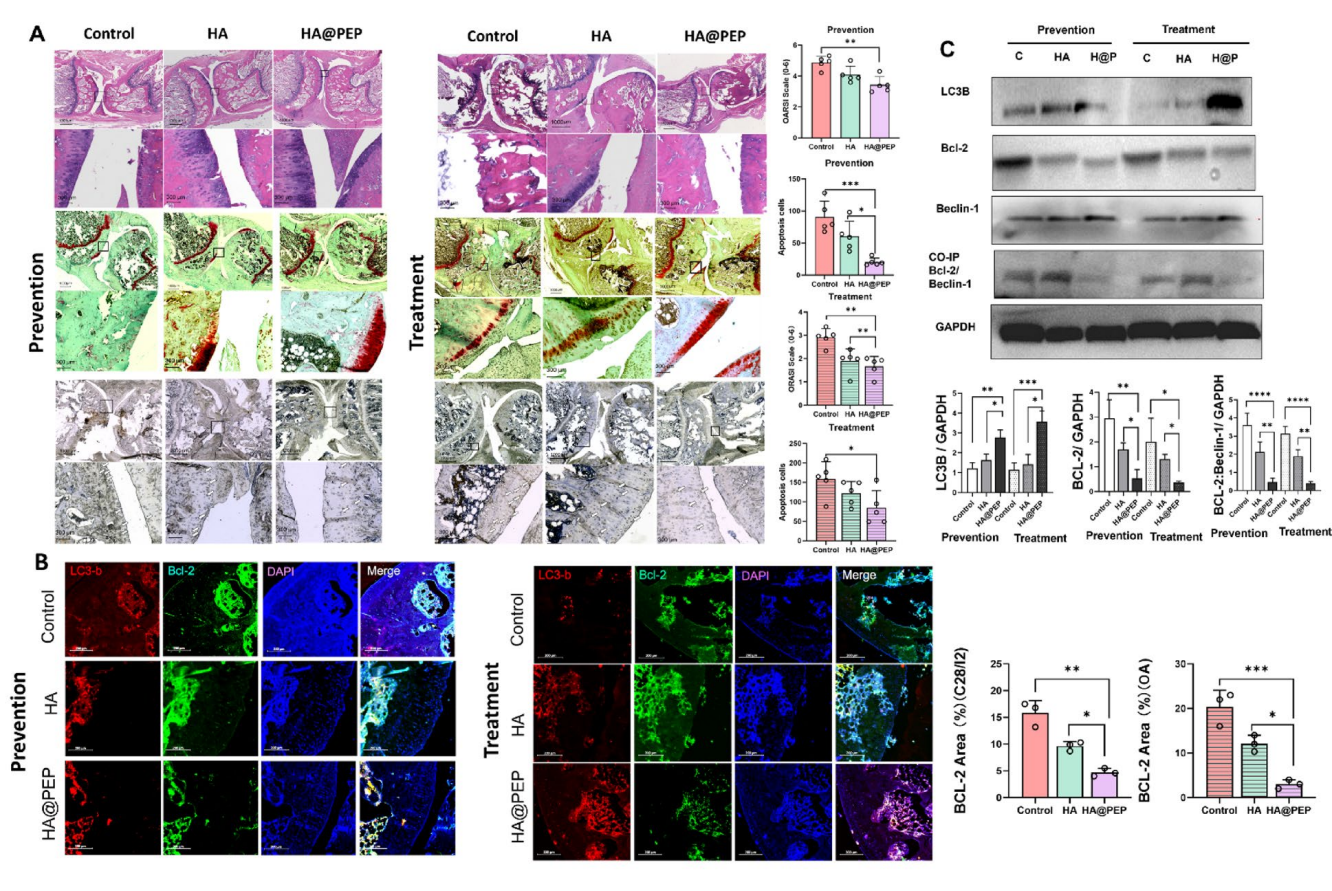
Histological, apoptotic, and molecular evaluation of articular cartilage in OA rats following HA@PEP treatment. **(A)** Hematoxylin and eosin (H&E), Safranin O–fast green, and TUNEL staining of knee joint sections from each group, showing cartilage morphology and chondrocyte apoptosis. **(B)** Immunofluorescence staining of rat cartilage showing LC3B (red) and BCL2 (green) expression in different treatment groups. **(C)** Western blot analysis of LC3B, BCL2, and BCL2–Beclin1 complex levels in joint tissues across groups. Data are presented as mean ± SD. *p < 0.05, **p < 0.01, ***p < 0.001, ****p < 0.0001

As expected, the combined ACLT + DMM procedure successfully induced osteoarthritic changes, consistent with previous reports [27]. In the prevention group, the HA@PEP-treated rats exhibited significantly higher OARSI scores compared to the control group, although no statistical difference was found when compared to the HA group. In the treatment group, the HA@PEP group showed significantly higher scores than both the control and HA groups, indicating superior cartilage preservation **(**Fig. 7A**)**.

TUNEL staining revealed a lower number of apoptotic chondrocytes in the HA@PEP group relative to the control and HA groups in the preventive setting. In the treatment group, the HA@PEP group also exhibited fewer apoptotic cells than the control group, although the difference with the HA group was not statistically significant **(**Fig. 7A**)**.

Immunofluorescence analysis demonstrated markedly reduced BCL2 protein levels in the HA@PEP group compared to both control groups under both preventive and therapeutic conditions **(**Fig. 7B**)**. Western blot results further confirmed these observations, showing elevated LC3B expression and a significant reduction in both BCL2 and BCL2–Beclin1 complex formation in the HA@PEP-treated rats, irrespective of treatment stage **(**Fig. 7C**)**. These protein-level findings were consistent with mRNA expression profiles obtained from qPCR analysis (Supplementary Fig. 7).

Together, these results suggest that HA@PEP administration enhances cartilage preservation by attenuating chondrocyte apoptosis and modulating autophagy via BCL2–Beclin1 signaling in both preventive and therapeutic contexts.

## Discussion

Purified Exosome Products (PEP), developed by the Mayo Clinic, have shown regenerative potential across multiple tissue types, including tendon, nerve, and skin [20–25, 34–37]. Preliminary in vitro studies have also suggested their ability to promote repair and suppress apoptosis in human chondrocytes [38]. However, the underlying mechanisms of their action in osteoarthritis (OA) remain to be fully elucidated.

In this study, we aimed to investigate the therapeutic mechanism of PEP in both preventing and treating OA. We explored its effects on autophagy and apoptosis in both immortalized and osteoarthritic human chondrocytes and validated these findings in a surgically induced rat OA model.

Our results demonstrated that PEP downregulates BCL2 gene expression and disrupts the inhibitory BCL2–Beclin1 interaction, thereby enhancing autophagy initiation while maintaining autophagic activity within a protective range. This modulation of the autophagy–apoptosis axis promotes cellular homeostasis by reducing programmed cell death and supporting chondrocyte survival. Collectively, these findings highlight the therapeutic potential of PEP as a disease-modifying nanotherapeutic for the management of osteoarthritis.

The interplay between autophagy and apoptosis represents a critical regulatory axis in cellular homeostasis. BCL2 is a key node in this pathway, functioning both as an inhibitor of autophagy—through binding to Beclin-1—and as a suppressor of apoptosis [39–41]. In our study, PEP treatment suppressed both the transcription and translation of BCL2, thereby relieving its inhibitory interaction with Beclin-1 and facilitating the initiation of autophagy. This controlled reduction in BCL2 levels allowed autophagy to be activated while remaining within a protective range, promoting cellular repair without triggering excessive autophagic activity or apoptosis.

**Fig. 8:**
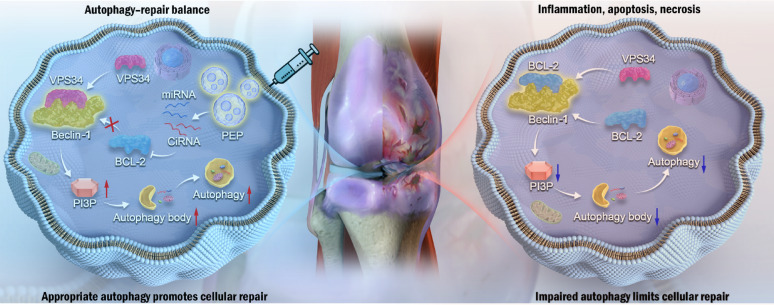
PEP restores autophagy–repair balance in osteoarthritis. PEP downregulates BCL-2, releases Beclin-1, and activates the VPS34–PI3P pathway to enhance autophagy and cellular repair. In contrast, impaired autophagy without PEP leads to reduced repair, inflammation, apoptosis, and necrosis. Interestingly, in C28/I2 cells, enhanced BCL2 expression did not significantly alter apoptosis levels, indicating a stronger apoptotic resistance in non-degenerative chondrocytes. This suggests that in normal cells, the anti-apoptotic effect of BCL2 overexpression may outweigh the negative impact of autophagy suppression. In contrast, OA chondrocytes, which are more vulnerable to stress-induced apoptosis, exhibit increased sensitivity to this autophagy-apoptosis imbalance

Notably, our data suggest that partial inhibition of BCL2 is sufficient to release Beclin-1 and initiate autophagy, while more substantial downregulation is required to affect apoptosis pathways [42–45]. This concept is illustrated in Fig. 8, where overexpression of BCL2 following PEP treatment suppressed autophagy and, in osteoarthritic (OA) chondrocytes, led to a modest increase in apoptosis.

Together, these findings underscore the importance of precise BCL2 modulation in balancing autophagic activity and apoptotic thresholds, supporting PEP’s potential as a fine-tuned regulator of joint tissue homeostasis.

The regulatory role of BCL2 in autophagy is inherently complex and context dependent. BCL2 has been shown to exert dual functions—either suppressing or promoting autophagy—depending on the cellular environment, the presence of co-regulatory molecules, and specific intracellular stress signals [46–48]. Other members of the BCL2 family, such as Bax and Bak, also interact with Beclin-1 and autophagy-related proteins, contributing to this dynamic balance through context-specific mechanisms.

A key regulatory node in autophagy initiation is the interaction between Beclin-1 and BCL2. While Beclin-1 forms a complex with Vps34 to initiate autophagosome formation, BCL2 can bind to Beclin-1 and inhibit this interaction, thereby suppressing autophagic activity [49–51]. This binding represents a critical checkpoint in autophagy regulation and underscores the need for precise modulation to maintain cellular homeostasis.

Maintaining autophagy within an optimal range is essential for eliminating damaged organelles, recycling cellular components, and preventing stress-induced apoptosis. However, excessive or insufficient autophagy can lead to deleterious outcomes, including autophagic cell death or unresolved cellular damage. In our study, PEP-induced autophagy was of moderate intensity—stronger than starvation-induced autophagy but less potent than that triggered by rapamycin. Notably, PEP-induced autophagy was associated with enhanced resistance to apoptosis, unlike rapamycin, which may induce excessive autophagy and lead to autophagic cell death. In contrast, starvation-induced autophagy appeared too weak to elicit reparative responses. These findings suggest that PEP modulates autophagy within a protective threshold, promoting cellular recovery without triggering harmful overactivation.

The ACLT + DMM model we employed in this study is less commonly used compared with single-lesion models such as DMM. Our rationale for selecting this dual-lesion approach was threefold. First, it better reflects the clinical scenario of post-traumatic OA, in which ligament rupture is frequently accompanied by meniscal injury. ACLT alone induces global joint instability with rapid cartilage degeneration, while DMM produces progressive, compartment-specific loss [27]. By combining the two, we aimed to mimic the synergistic effects of instability and altered load distribution. Second, dual-lesion models have been shown to accelerate and intensify OA progression. In larger animals, ACLT combined with medial meniscectomy (ACLT + MMx) produced more consistent and severe phenotypes in a shorter timeframe compared with either procedure alone [28, 29]. Third, this model provided a stringent setting to evaluate disease-modifying interventions, as it induces both structural degeneration and pain-related behaviors, making it a robust preclinical stress test. We acknowledge that ACLT + DMM represents a relatively severe form of joint injury, which may limit direct extrapolation to slowly progressive OA. However, we considered this model appropriate for evaluating the therapeutic potential of PEP, given its ability to generate consistent and reproducible pathological changes [52]. 

In addition, it is relevant to compare PEP with MSC-derived exosomes, which are the most extensively studied EVs in OA. Mesenchymal stem cell (MSC)–derived exosomes are among the most extensively studied extracellular vesicle–based therapies for OA and have demonstrated chondroprotective, anti-inflammatory, and immunomodulatory properties in multiple preclinical models [53–55]. For example, bone marrow MSC-exosomes have been reported to deliver regulatory miRNAs to cartilage, thereby balancing extracellular matrix synthesis and degradation [54], and MSC-EVs have also been shown to exert beneficial effects on cartilage, subchondral bone, and synovial tissues [56]. Nonetheless, their clinical translation faces challenges, including donor-to-donor variability, heterogeneous culture conditions, and difficulties in large-scale manufacturing, all of which contribute to batch-to-batch inconsistency [57]. In contrast, PEP is derived from pooled human plasma and produced under GMP conditions using a standardized process, yielding a stable and reproducible product with defined QC specifications. This confers advantages in scalability, product consistency, and safety for clinical application. Although a head-to-head comparison was beyond the scope of the present study, future work directly comparing PEP with MSC-derived exosomes will be valuable to delineate their relative therapeutic efficacy in OA.

Despite the promising results, several limitations should be acknowledged. The dualistic nature of BCL2 in autophagy regulation requires further mechanistic exploration. Although our findings suggest that PEP downregulates BCL2 transcription and alleviates its inhibitory interaction with Beclin-1, the exact molecular pathways by which exosomes mediate this regulatory effect remain unclear. Future studies should focus on elucidating the broader signaling networks involved in BCL2-mediated autophagy modulation and the role of exosomal cargo in this process. Although our findings indicate that PEP elicits a moderate autophagic response that is cytoprotective, a systematic dose–response analysis of PEP on autophagy (e.g., LC3B) and apoptosis (e.g., caspase-3/7) markers would provide further mechanistic insight and help delineate the therapeutic window of PEP-induced autophagy.

Beyond mechanistic considerations, an important aspect for the clinical translation of extracellular vesicle–based therapeutics is batch-to-batch reproducibility. In the present study, we employed a single GMP-produced lot of PEP (#23001A). To further evaluate product consistency, we compiled a comparative QC summary (Supplementary Table 2) including data from previous investigations in ischemic wound healing, rotator cuff tendon-bone healing, and tendon explant repair. Across these independent studies, PEP consistently demonstrated typical exosomal morphology (~ 100 nm by TEM), particle size distributions within the expected range (mode ~ 60–110 nm, D50 ~ 80–95 nm, D90 ~ 150–180 nm), particle concentrations in the order of 10^8^–10^11^ particles/mL, and robust expression of exosomal markers such as CD9 and CD63. These findings collectively indicate that the GMP process yields a stable and reproducible clinical-grade product, reinforcing the robustness and translational potential of PEP. Together with the consistent efficacy observed across different models, these QC data support the stability of PEP as a reproducible clinical-grade product.

The regulatory mechanism through which PEP influences the BCL2–Beclin-1 axis has yet to be fully delineated. As carriers of diverse RNA species and proteins, exosomes are capable of modulating intracellular signaling pathways. One potential mode of action involves PEP-delivered microRNAs that directly or indirectly target BCL2 mRNA, thereby suppressing its transcription and translation. For instance, miR-15/16 and miR-34 family members are known to bind the 3′UTR of BCL2 and inhibit its expression [58, 59], whereas miR-140 and miR-146a have been implicated in cartilage homeostasis and autophagy regulation [60–62]. Supporting this hypothesis, our small-RNA sequencing data from an ongoing study identified several microRNAs enriched in PEP—including miR-16-5p, miR-151a-3p, miR-223-3p, let-7b-5p, and miR-146a-5p—which are computationally predicted to interact with the BCL2 regulatory network. Concurrently, exosomal proteins such as heat shock proteins (HSPs) may facilitate stress-adaptive signaling, indirectly promoting Beclin-1 dissociation from BCL2 [63].

A recent study from Mayo Clinic Florida further revealed that the therapeutic efficacy of PEP in viral myocarditis is closely associated with its biomolecular cargo, as evidenced by integrated miRNA and proteomic profiling [64]. These independent findings reinforce the notion that PEP-derived components can mediate transcriptional repression and pathway-level modulation. Thus, the downregulation of BCL2 observed in our system may result from the combined action of PEP-carried small RNAs and proteins operating at multiple regulatory tiers. While the present study establishes the functional outcome of PEP on the BCL2–Beclin-1 axis, subsequent integrated omics approaches will be necessary to systematically identify the specific exosomal constituents responsible for this effect. While the present study relied on C28/I2 cells and OA primary chondrocytes, which are widely used for mechanistic studies, future investigations should incorporate advanced systems such as 3-D pellet cultures or iPSC-derived chondrocytes. These models more closely recapitulate the native cartilage microenvironment and will provide additional validation of the translational potential of PEP.

Another limitation is that baseline gait parameters were only collected for the prevention cohort; in the treatment cohort, animals were randomized at 8 weeks post-surgery, when OA pathology was established, and both histological and functional readouts confirmed comparable OA severity across groups. Finally, although our in vitro release assays and functional outcomes suggest sustained intra-articular activity of PEP, future studies using biodistribution methods (e.g., IVIS imaging or LC-MS tracking of labeled exosomes) will be required to precisely define its pharmacokinetics and long-term retention.

In-depth mechanistic analyses—including time-course studies, single-cell resolution approaches, and multi-omics profiling—will be crucial for dissecting the interplay between autophagy and apoptosis. These efforts will advance our understanding of PEP’s therapeutic potential and support its further development as a disease-modifying nanotherapeutic for osteoarthritis prevention and treatment.

These findings highlight PEP’s ability to downregulate BCL2 transcription, thereby modulating the BCL2–Beclin-1 axis and promoting protective autophagy. This mechanistic insight offers a promising foundation for the development of exosome-based strategies in osteoarthritis therapy.

## Conclusion

This study demonstrates that a clinically applicable purified exosome product (PEP) holds strong therapeutic potential for both the prevention and treatment of osteoarthritis. Mechanistically, PEP downregulates BCL2 transcription and disrupts the BCL2–Beclin-1 interaction, thereby promoting protective autophagy and reducing chondrocyte apoptosis. These findings provide new insight into the autophagy–apoptosis regulatory axis and establish a foundation for future exploration of exosome-based disease-modifying strategies in osteoarthritis management.

## Supplementary Information

Supplementary Material 1

Supplementary Material 2

Supplementary Material 3

Supplementary Material 4

Supplementary Material 5

Supplementary Material 6

Supplementary Material 7

Supplementary Material 8

Supplementary Material 9

Supplementary Material 10

Supplementary Material 11

## Data Availability

The datasets used and analyzed during the current study are available from the corresponding author on reasonable request.

## Abbreviations

OA: Osteoarthritis
PEP: Purified Exosome Product
HA: Hyaluronic Acid
ACLT: Anterior Cruciate Ligament Transection
DMM: Destabilization of the Medial Meniscus
BCL-2: B-cell lymphoma 2
PCR: Polymerase Chain Reaction
WB: Western Blot
Co-IP: Co-immunoprecipitation
LC3: Microtubule-associated protein 1 A/1B-light chain 3
GAPDH: Glyceraldehyde 3-phosphate dehydrogenase
H&E: Hematoxylin and Eosin
OARSI: Osteoarthritis Research Society International
TUNEL: Terminal deoxynucleotididyl transferase dUTP nick end labeling
3-MA: 3-Methyladenine
PBS: Phosphate-Buffered Saline
FBS: Fetal Bovine Serum
DMEM: Dulbecco’s Modified Eagle Medium
CT: Computed Tomography
SEM: Scanning Electron Microscope
RNA: Ribonucleic Acid
cDNA: Complementary DNA
HRP: Horseradish Peroxidase
DAPI: 4’,6-diamidino-2-phenylindole
API: Advanced Product Incubator
IRB: Institutional Review Board
