# Supplementary figures and images for: A therapeutic-grade purified exosome system alleviates osteoarthritis by regulating autophagy through the BCL2–Beclin1 axis
Source: J Nanobiotechnology. 2025 Dec 5;24:31. doi: 10.1186/s12951-025-03807-y (PMC12797455; doi:10.1186/s12951-025-03807-y)

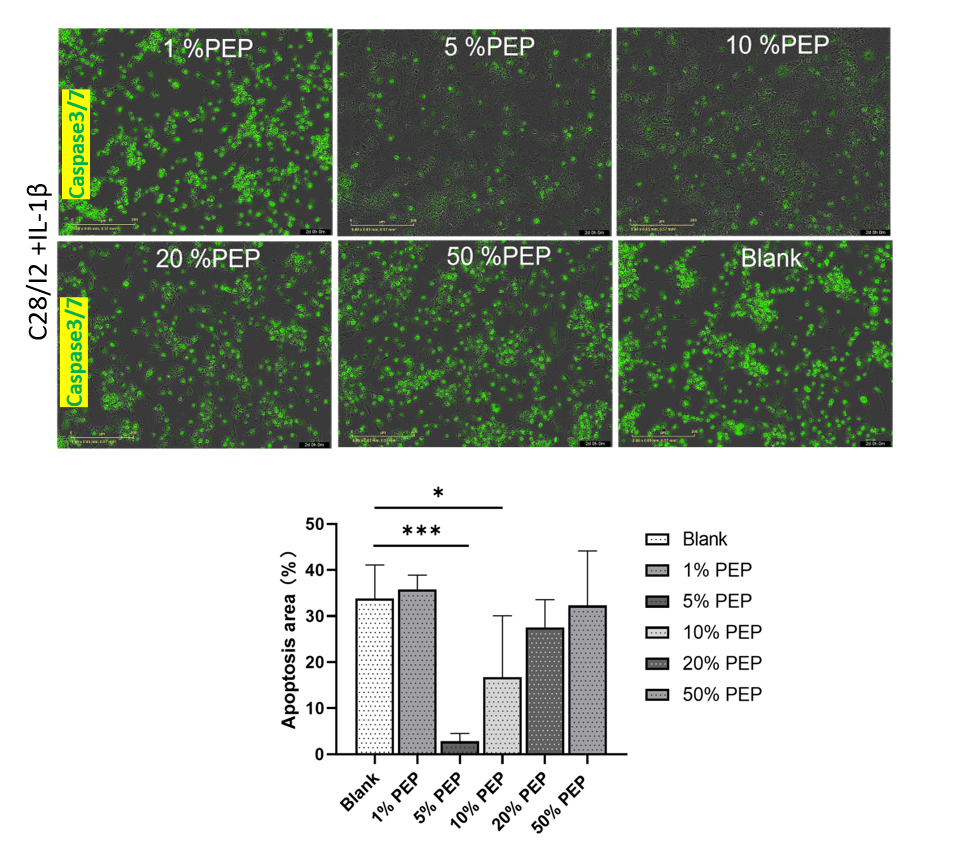

Supplement: Supplementary file 1 — Supplementary Material 1 [file 12951_2025_3807_MOESM1_ESM.tif]

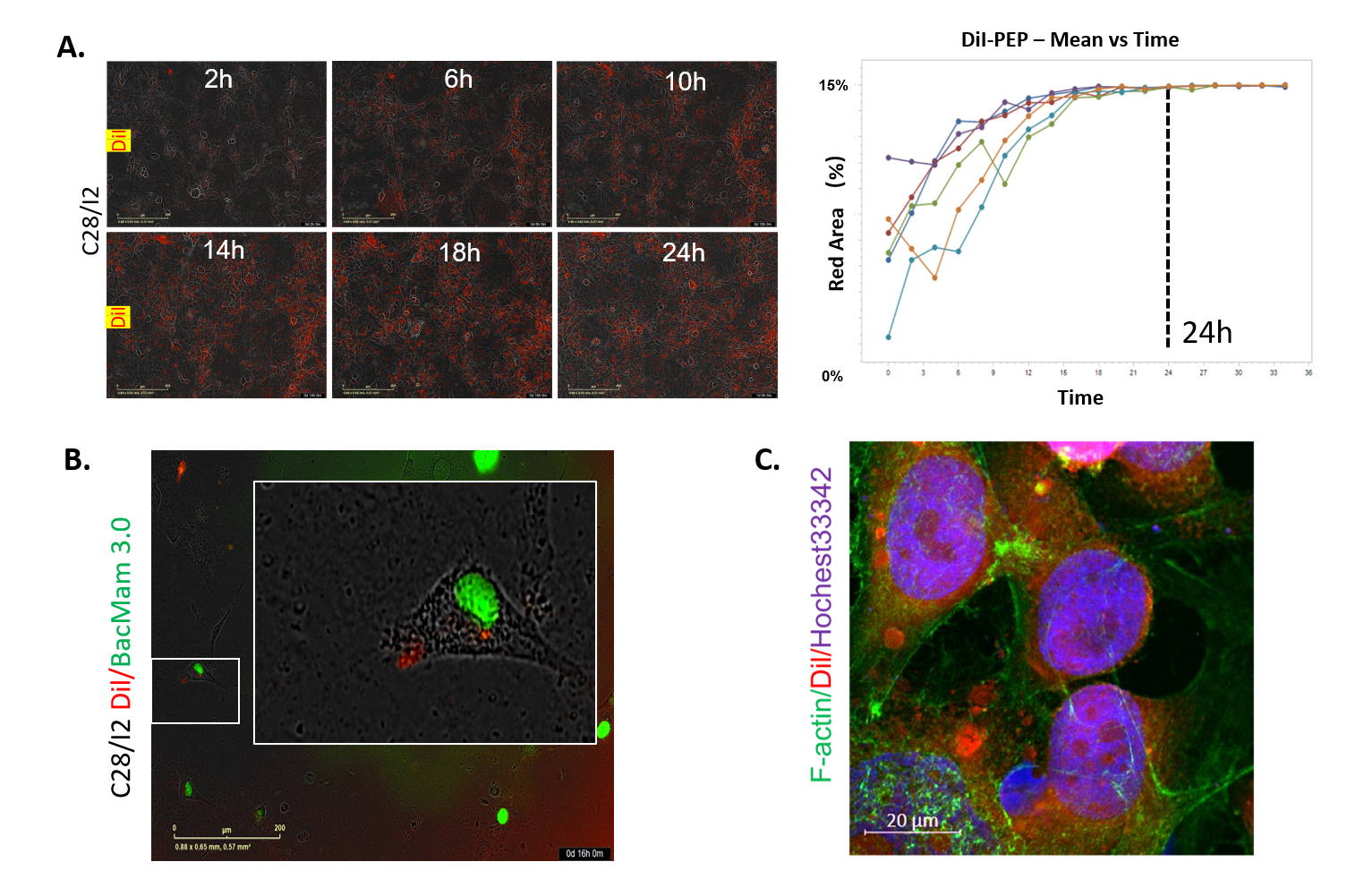

Supplement: Supplementary file 2 — Supplementary Material 2 [file 12951_2025_3807_MOESM2_ESM.tif]

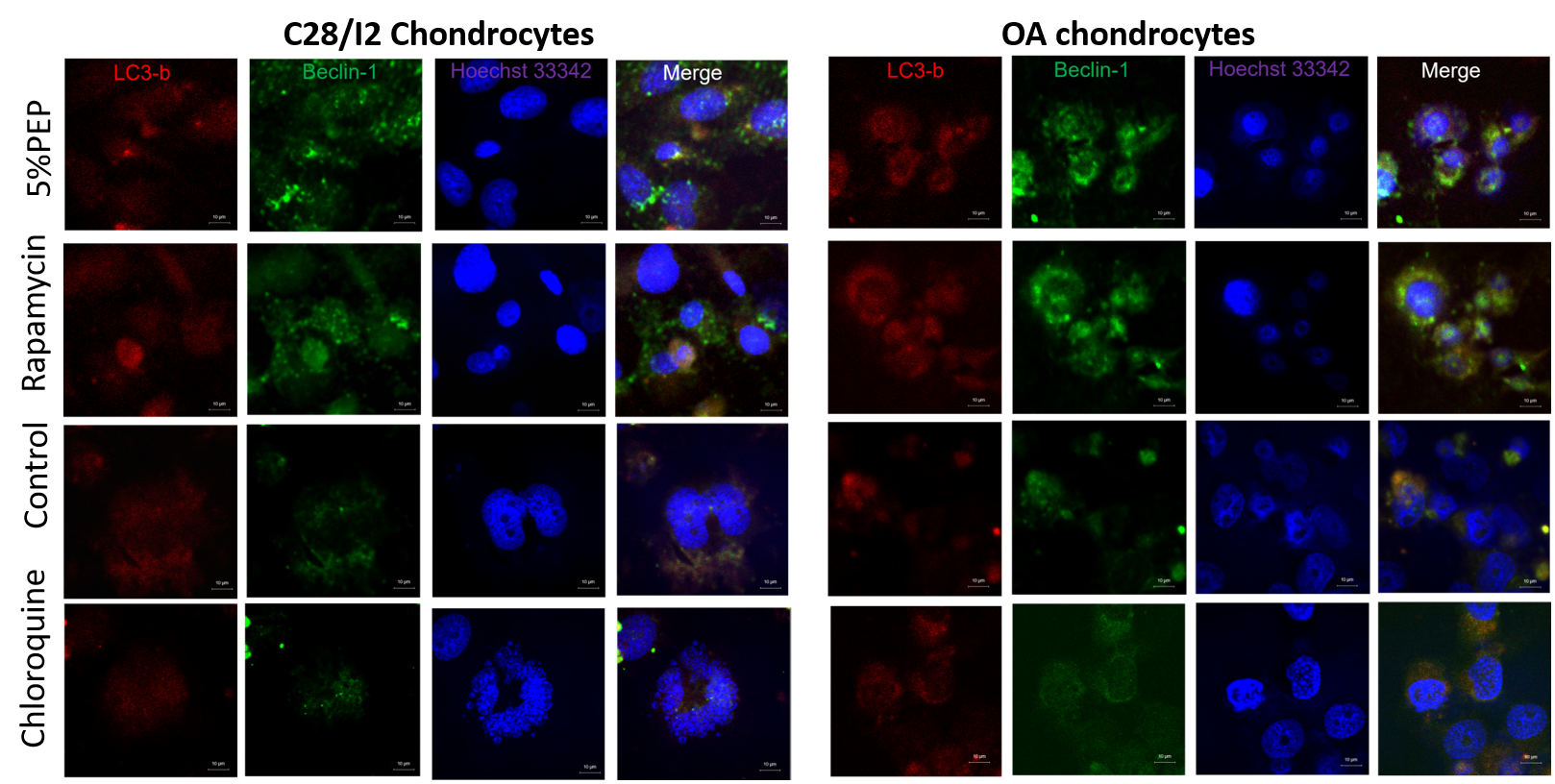

Supplement: Supplementary file 3 — Supplementary Material 3 [file 12951_2025_3807_MOESM3_ESM.tif]

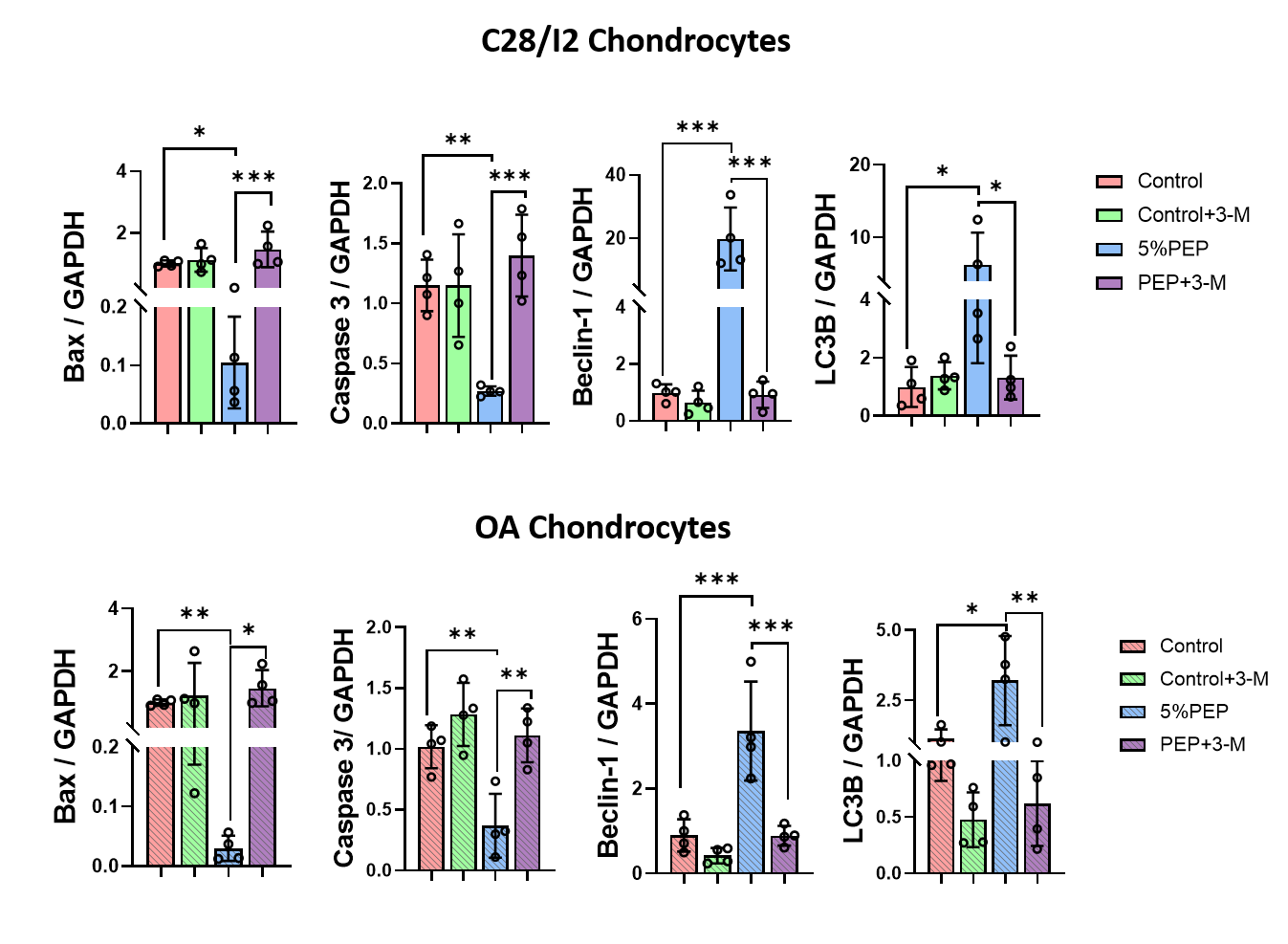

Supplement: Supplementary file 4 — Supplementary Material 4 [file 12951_2025_3807_MOESM4_ESM.tif]

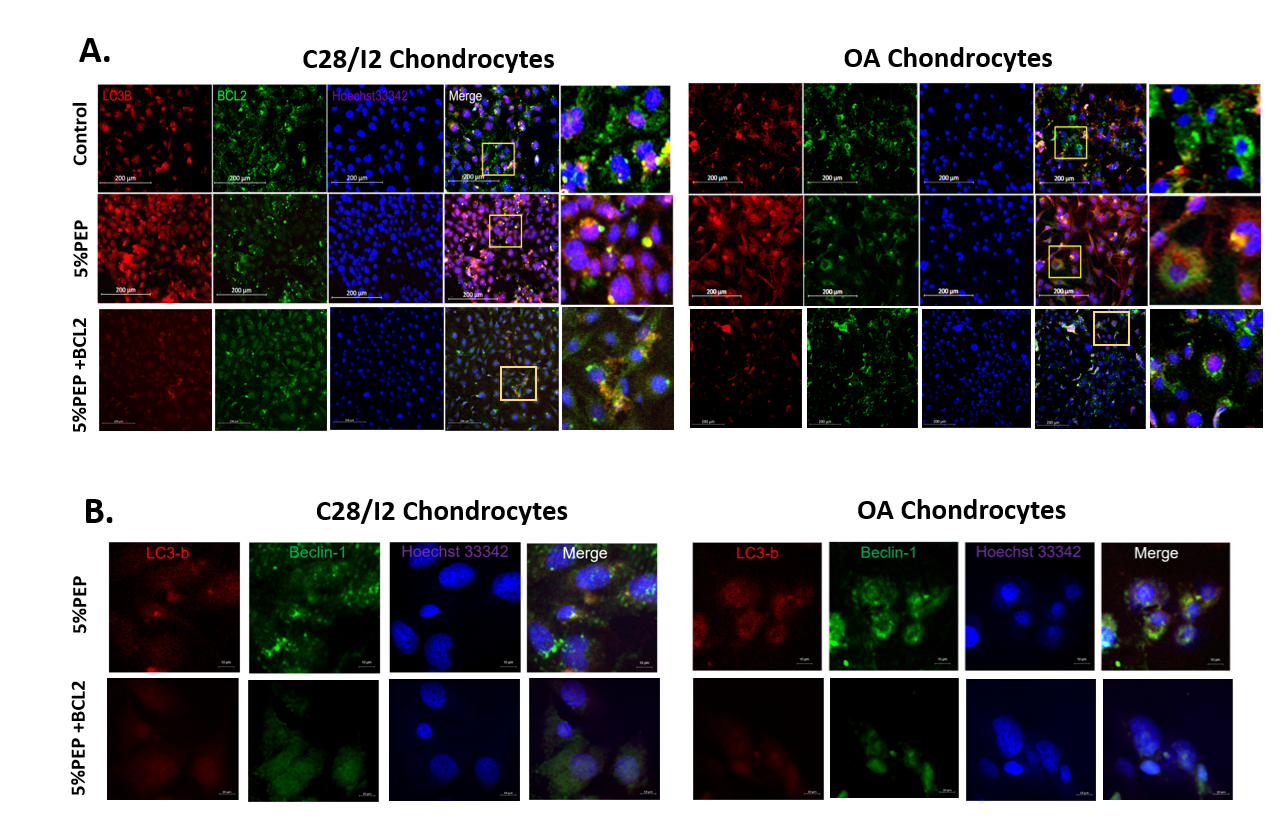

Supplement: Supplementary file 5 — Supplementary Material 5 [file 12951_2025_3807_MOESM5_ESM.tif]

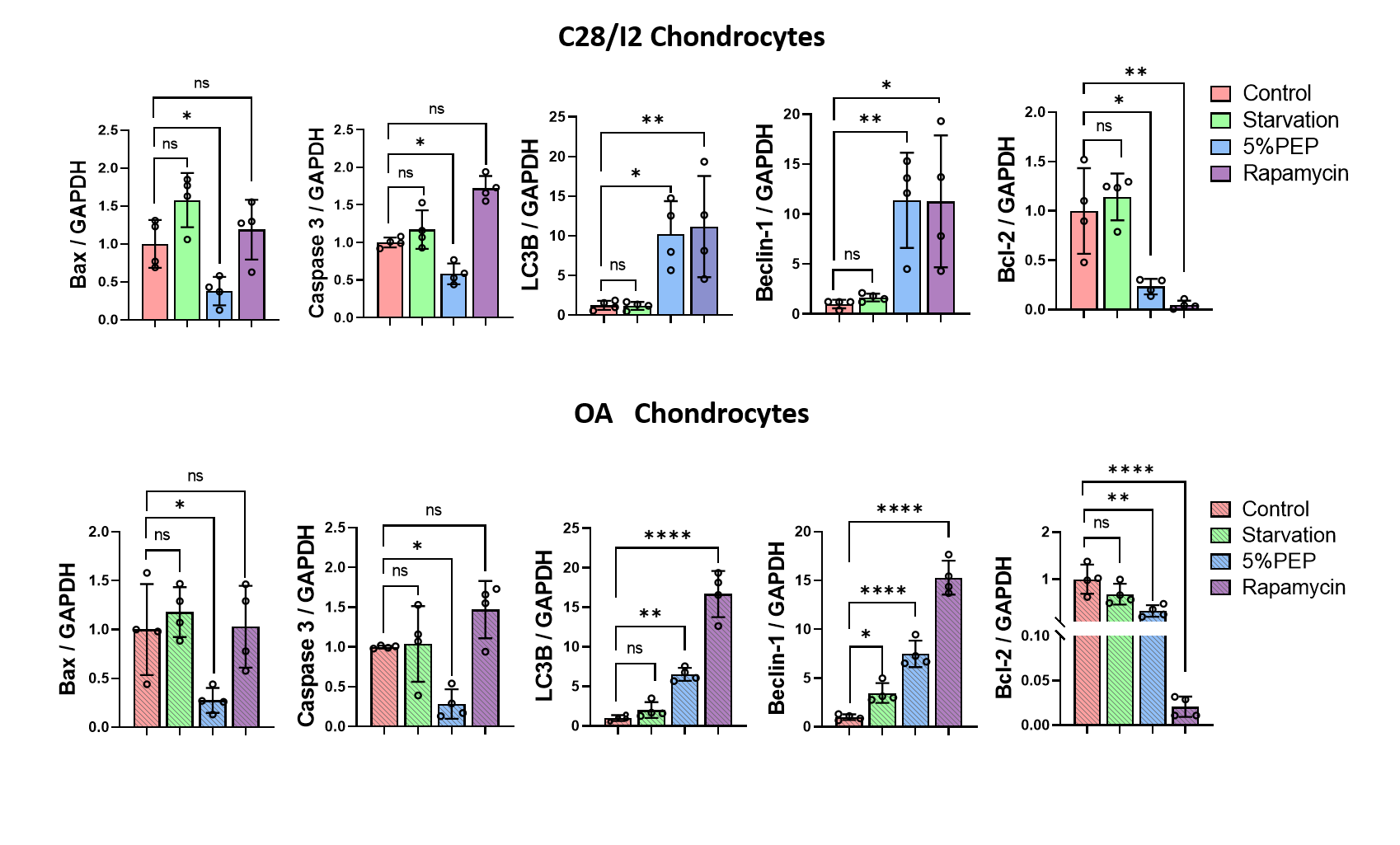

Supplement: Supplementary file 6 — Supplementary Material 6 [file 12951_2025_3807_MOESM6_ESM.tif]

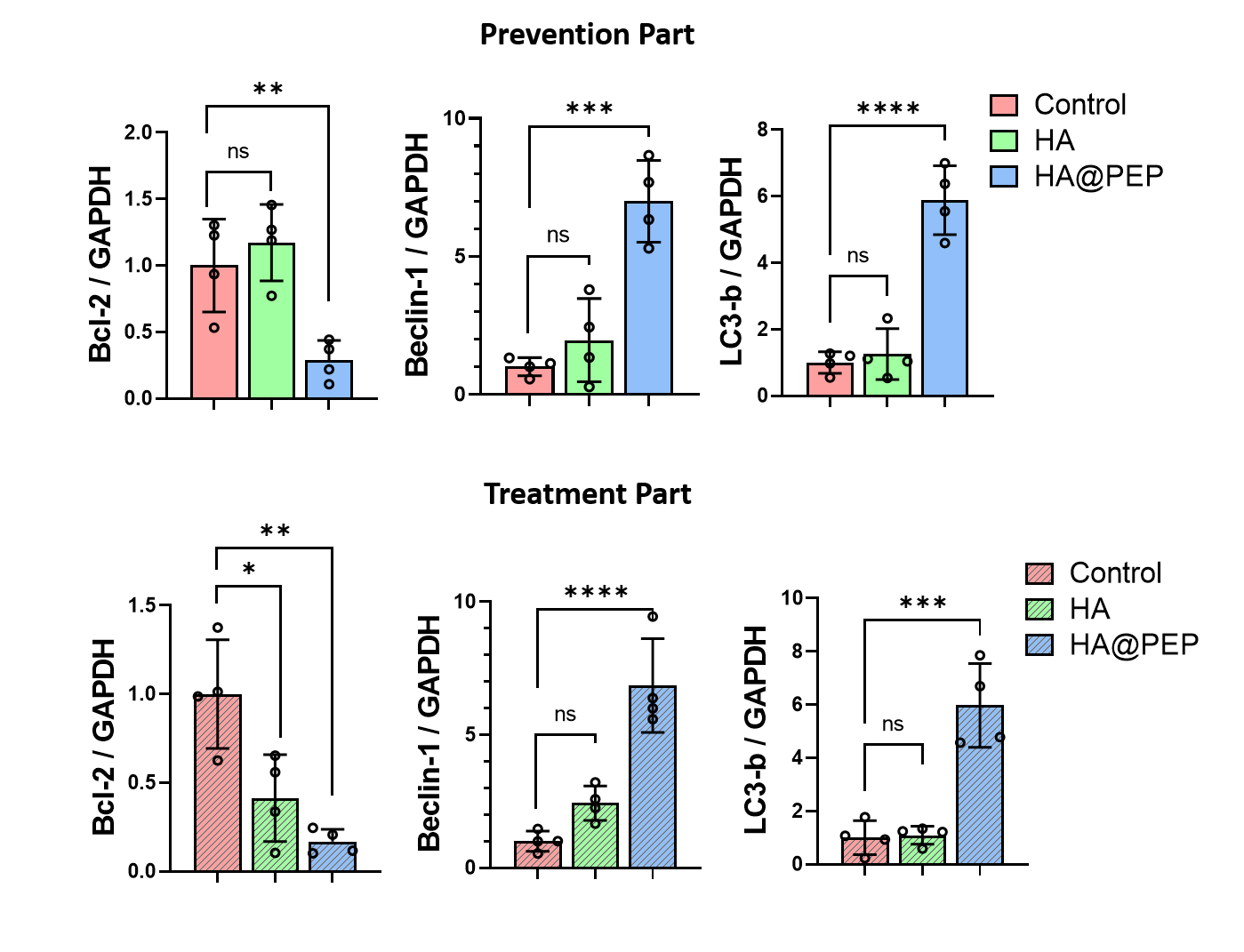

Supplement: Supplementary file 7 — Supplementary Material 7 [file 12951_2025_3807_MOESM7_ESM.tif]

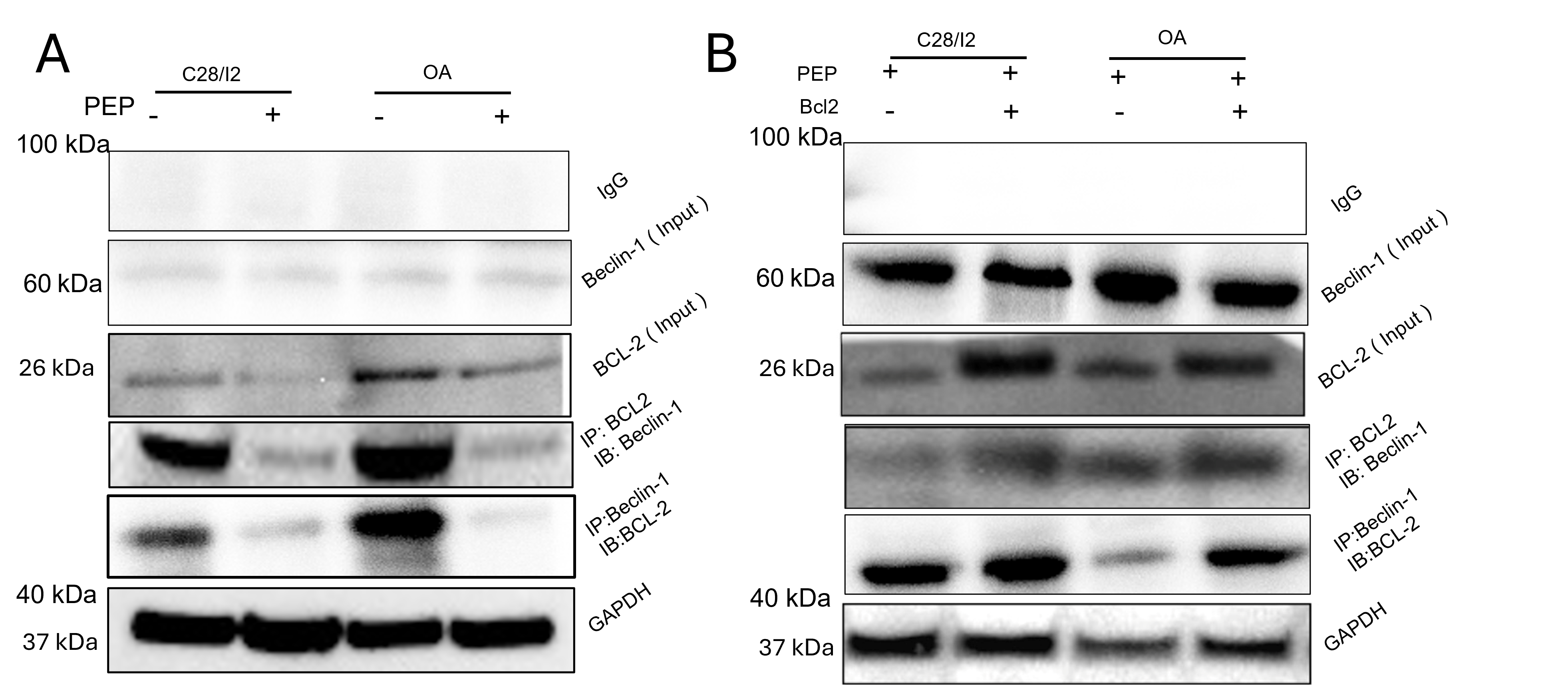

Supplement: Supplementary file 11 — Supplementary Material 11 [file 12951_2025_3807_MOESM11_ESM.tif]
