# Supplementary material for: A therapeutic-grade purified exosome system alleviates osteoarthritis by regulating autophagy through the BCL2–Beclin1 axis
Source: J Nanobiotechnology. 2025 Dec 5;24:31. doi: 10.1186/s12951-025-03807-y (PMC12797455; doi:10.1186/s12951-025-03807-y)
Supplement: Supplementary file 8 — Supplementary Material 8 [file 12951_2025_3807_MOESM8_ESM.docx]

Table 1：Primers

| Human | | |
| --- | --- | --- |
| LC3B | Forward: 5’- GAGAAGCAGCTTCCTGTTCTGG -3’ | |
|  | Reverse: 5’- GTGTCCGTTCACCAACAGGAAG -3’ | |
| Caspase 3 | Forward: 5’- TGGTTCATCCAGTCGCTTTGT -3’ | |
|  | Reverse: 5’- CCCGGGTAAGAATGTGCATAAA -3’ | |
| Beclin-1 | Forward: 5- GTAGTACTGCCACGTCCTCA-3 | |
|  | Reverse: 5- GGTTCTTCCCTGACTTCTCCT-3 | |
| Bcl-2 | Forward: 5- ATCGCCCTGTGGATGACTGAGT-3 | |
|  | Reverse: 5- GCCAGGAGAAATCAAACAGAGGC-3 | |
| Bax | Forward: 5- TCAGGATGCGTCCACCAAGAAG -3 | |
|  | Reverse: 5- TGTGTCCACGGCGGCAATCATC -3 | |
| GAPDH | Forward: 5′-ACCCAGAAGACTGTGGATGG -3′ | |
|  | Reverse: 5′-CACATTGGGGGTAGGAACAC -3′ | |
| Rats | | |
| LC3B | | Forward: 5’- ATGGTAGTCTGTGGTGGTGG -3’ |
|  |  | Reverse: 5’- CATACCTTGAATCCCAGCGC-3’ |
| Caspase 3 | | Forward: 5′-GTGGAACTGACGATGATATGGC-3′ |
|  |  | Reverse: 5′-CGCAAAGTGACTGGATGAACC-3′ |
| Bcl-2 | | Forward: 5- AGTCTCAAGCTCATCGGACC-3 |
|  |  | Reverse: 5- GGGAGGCGGACTATAGGTTC-3 |
| Bax | | Forward: 5- ATGTAGATGTGGGCAGTGCT-3 |
|  |  | Reverse: 5- GCCCAGACACCTAGTTCCTT-3 |
| Beclin-1 | | Forward: 5- ACTGGACACGAGCTTCAAGA-3 |
|  |  | Reverse: 5- CTCCTTCCCTCTGCAGACAA-3 |
| GAPDH | | Forward: 5′- CAAAATGGCTCCGGTGGTAG -3′ |
|  |  | Reverse: 5′- CTGGTTTCTGGAGGATGGGT -3′ |
