# Supplementary material for: A therapeutic-grade purified exosome system alleviates osteoarthritis by regulating autophagy through the BCL2–Beclin1 axis
Source: J Nanobiotechnology. 2025 Dec 5;24:31. doi: 10.1186/s12951-025-03807-y (PMC12797455; doi:10.1186/s12951-025-03807-y)
Supplement: Supplementary file 9 — Supplementary Material 9 [file 12951_2025_3807_MOESM9_ESM.docx]

# Supplementary Table 2. Comparison of QC Characteristics of PEP Across Studies and Batches

| QC Metric | This study (Lot #23001A) | Wound healing study (TGF-β, 2020) | Rotator cuff healing study (2022) | Tendon explant patch study (2023) |
| --- | --- | --- | --- | --- |
| Particle morphology (TEM) | Typical spherical vesicles (~100 nm) | Typical spherical vesicles (50–150 nm) | Typical spherical vesicles (~100 nm) | Typical spherical vesicles (~100 nm) |
| Particle concentration (NTA) | 1.2 × 10^11 particles/mL | ≈1.0 × 10^11 particles/mL | 1.9 × 10^8 ± 1.5 × 10^7 particles/mL | 4.68 × 10^8 ± 6.57 × 10^6 particles/mL |
| Exosomal markers (WB) | CD9, CD63 positive (~1:1) | CD9, CD63 positive (~1:1) | Verified by TEM & NTA | Verified by TEM & NTA |
| Reference | This study | TGF-β loaded exosome enhances ischemic wound healing in vitro and in vivo | Effects of purified exosome product on rotator cuff tendon-bone healing in vitro and in vivo | A novel engineered purified exosome product patch for tendon healing: An explant in an ex vivo model |

Note: QC characteristics across studies demonstrate consistent morphology, particle size, concentration, and marker expression, indicating that PEP is a stable and reproducible clinical-grade product across different batches and applications.
