# Supplementary material for: A therapeutic-grade purified exosome system alleviates osteoarthritis by regulating autophagy through the BCL2–Beclin1 axis
Source: J Nanobiotechnology. 2025 Dec 5;24:31. doi: 10.1186/s12951-025-03807-y (PMC12797455; doi:10.1186/s12951-025-03807-y)
Supplement: Supplementary file 10 — Supplementary Material 10 [file 12951_2025_3807_MOESM10_ESM.docx]

**Supplementary Figure.1: A:** Animal experiment grouping and timeline. **B:** Surgical procedure involving medial meniscectomy after arthrotomy of the right knee. **C:** Surgical procedure involving anterior cruciate ligament dissection of the right knee. **D:** Ultrasound-guided injection into the joint cavity of the right knee. **E:** Ultrasound-monitored drug injection into the joint cavity. **F**: Nano Sight analysis depicting the quantity and size distribution of PEP particles. **G:** Characterization of Exosome-Associated Proteins. **H:** Visualization of PEP particles using scanning electron microscope, with yellow arrows indicating PEP particles. **I:** Release experiments demonstrating the daily release of PEP particles in a 50% HA@PEP solution over the course of one week.

**Supplementary Figure.2：**To assess chondrocyte apoptosis, we conducted co-incubation experiments with varying concentrations of PEP and C28/I2 chondrocytes, all in the presence of 10 ng/mL IL-1β, for a duration of 48 hours. In our visualization, Caspase3/7 protein is represented by the green color. Notably, statistical significance is denoted as follows: (* p<0.05, ** p<0.001, *** p<0.0001).

**Supplementary Figure.3：A:** Using IncuCyte monitoring, we observed the uptake of 5% PEP by C28/I2 chondrocytes over a 24-hour period. The PEP, stained with Dil, is visualized in red. **B:** At the 16-hour mark, we captured another image depicting PEP uptake by chondrocytes. In this image, Dil-stained PEP is shown in red, while chondrocytes are stained green using BacMam 3.0. **C:** After 24 hours, we analyzed the distribution of PEP within chondrocytes.

**Supplementary Figure.4：**The Autophagy Flow Assay employs distinct color representations: red signifies the presence of LC3-b protein, green indicates the presence of beclin-1 protein, purple denotes Hoechest33342, while white represents the fusion of these colors.

**Supplementary Figure.5：**We assessed the expression of autophagy and apoptosis-related genes in both C28/I2 and osteoarthritic (OA) chondrocytes, both in the presence and absence of the autophagy inhibitor 3-methyladenine. “3-M” means 3-methyladenine. (* p<0.05, ** p<0.001, *** p<0.0001).

**Supplementary Figure.6：A**: Evaluation of LC3-b protein and Bcl-2 protein expression in both control and 5% pep-treated groups, alongside the impact following the introduction of the Bcl2 gene after 5% pep exposure. LC3B protein is depicted in red, Bcl2 protein in green, Hoechst33342 in purple, and the fusion of these colors is represented in white. **B**: Analysis of alterations in autophagy flux in response to 5% pep exposure, and subsequent changes observed after the introduction of the Bcl2 gene following exposure to 5% pep. LC3B protein is visualized in red, Becline-1 protein in green, Hoechst33342 in purple, and the fusion of these hues is depicted in white.

**Supplementary Figure.7**: Gene expression profiling of apoptosis and autophagy-related genes in response to three distinct autophagy induction stimuli: control (absence of autophagy inducers), rapamycin treatment, and 5% PEP intervention. (* p<0.05, ** p<0.001, *** p<0.0001).

**Supplementary Figure.8**: IgG was included as a negative control. Input lanes confirm the presence of BCL-2 (~26 kDa) and Beclin-1 (~60 kDa) in total lysates. Both forward (IP: Beclin-1; IB: BCL-2) and reverse (IP: BCL-2; IB: Beclin-1) Co-IP are shown. GAPDH (~37 kDa) served as a loading control.
